# Membranes Decouple α‑Synuclein Surface Assembly and Kinetic Restriction from Persistent Amyloid-like Intermolecular Organization

**DOI:** 10.1021/jacsau.6c01222

**Published:** 2026-09-24

**Authors:** Jinyu Chen, Xiakun Chu

**Affiliations:** † Advanced Materials Thrust, Function Hub, 567841 The Hong Kong University of Science and Technology (Guangzhou), Guangzhou, Guangdong 511453, China; ‡ Guangzhou Municipal Key Laboratory of Materials Informatics, The Hong Kong University of Science and Technology (Guangzhou), Guangzhou, Guangdong 511453, China; § Division of Life Science, The Hong Kong University of Science and Technology, Clear Water Bay, Kowloon, Hong Kong SAR 999077, China

**Keywords:** α-synuclein, membrane-associated condensates, lipid membranes, amyloid-like intermolecular organization, coarse-grained simulations

## Abstract

Membrane association places α-synuclein (αSyn) at the boundary between physiological surface organization and pathological amyloid assembly, supporting vesicle clustering, surface condensation, and amyloid nucleation. How interfacial recruitment, condensate cohesion, and persistent amyloid-like organization are coupled or decoupled remains unclear. Because membrane binding also induces helical ordering in the N-terminal membrane-binding region and the aggregation-prone non-amyloid-β component (NAC) region, we used multichain coarse-grained simulations to separate conformational ordering from recruitment by an anionic POPC/POPG bilayer. Helical/ordered segments promoted locally dense, directionally packed assemblies but weakened global condensate cohesion. Membrane association produced flattened surface assemblies and increased the protein population outside the largest condensate. Despite becoming dynamically restricted, these membrane-associated condensates did not show systematically prolonged NAC contact lifetimes. Thus, membrane-driven surface assembly and kinetic restriction can be decoupled from persistent amyloid-like intermolecular organization, explaining how lipid interfaces promote αSyn clustering without necessarily driving amyloid-competent organization.

## Graphical abstract

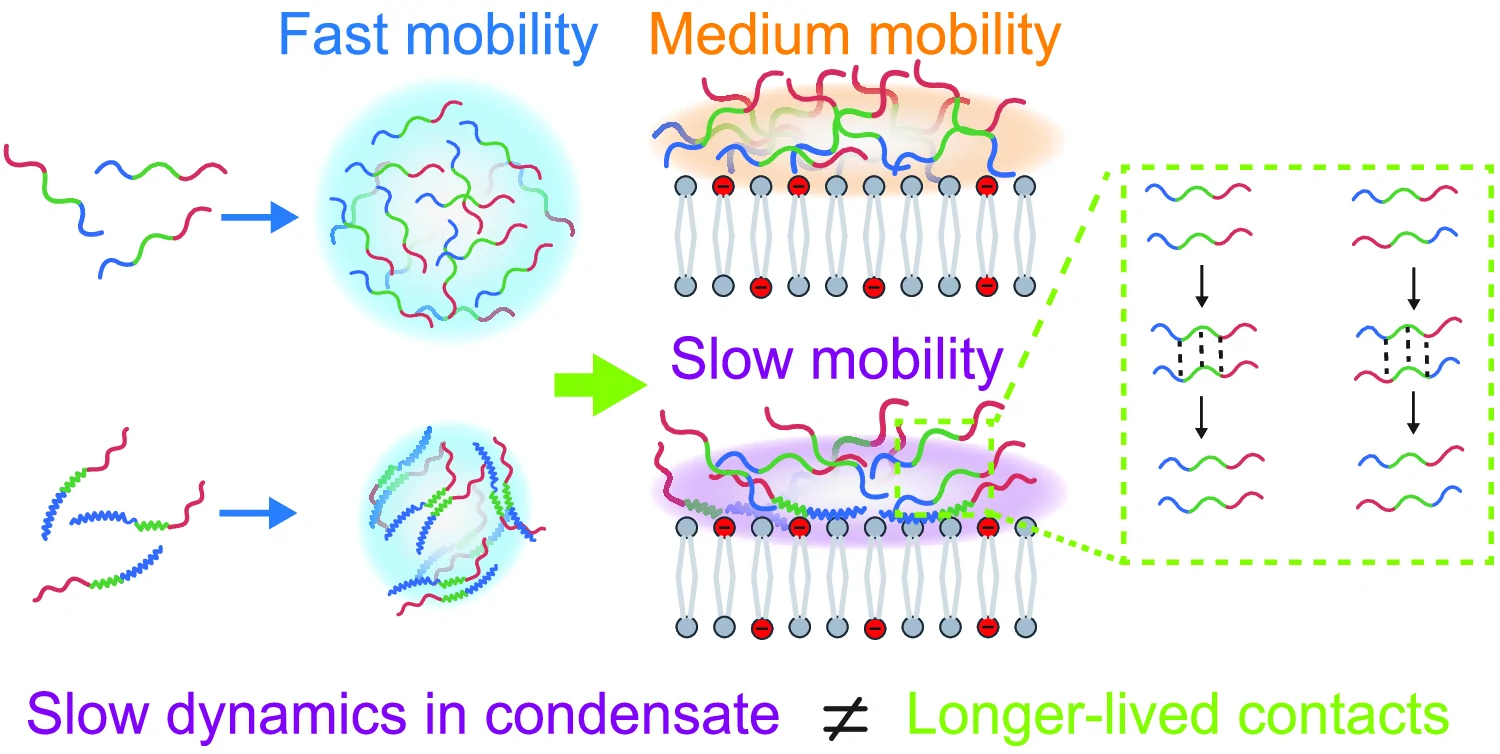

## 1 Introduction

α-Synuclein (αSyn) is a presynaptic intrinsically disordered protein whose assembly at lipid interfaces lies at the boundary between physiological membrane-associated organization and pathological amyloid formation.^1–4^ In neurons, αSyn dynamically associates with synaptic vesicles and other anionic membrane surfaces, where its N-terminal region mediates membrane recognition and helix formation.^3–5^ Through these membrane-associated interactions, αSyn contributes to vesicle clustering, vesicle trafficking, SNARE-complex assembly, and synaptic regulation.^2,6–8^ In Parkinson’s disease and related synucleinopathies, however, αSyn assembles into amyloid-rich aggregates that constitute major components of Lewy bodies.^1,9,10^ Cryo-electron tomography further showed that Lewy pathology contains crowded organelles and lipid membranes in addition to fibrillar material.^11^ This dual behavior makes membrane-associated αSyn assembly a state-selection problem: membrane surfaces can recruit and organize αSyn for physiological function, but they can also create locally enriched environments from which pathological self-assembly may emerge.^12–15^

A central unresolved question is why membrane recruitment does not lead to a single, uniform assembly outcome. Lipid interfaces can enrich αSyn and support divergent assembly outcomes, ranging from self-limiting membrane-bound multimerization to primary nucleation and fibrillation under appropriate conditions.^12,15^ Recent work has shown that a membrane interfacial potential can drive αSyn surface condensation at subcritical bulk concentrations through a prewetting-like mechanism, with membrane-associated condensates proposed to act as nucleation sites for fibril formation.^16^ More broadly, membrane surfaces can organize biomolecular phase separation by coupling interfacial wetting, electrostatics, lateral confinement, and membrane-coupled phase behavior.^17–20^ These studies establish membrane-mediated enrichment as an important upstream regulator of αSyn assembly. However, local enrichment at the membrane surface does not necessarily imply either the formation of a cohesive condensate or the development of persistent amyloid-like intermolecular organization.

The broader distinction between assembly and amyloid maturation is also evident in membrane-free systems: mass-photometry measurements identified dynamic nanoscale clusters at physiologically relevant, subsaturated concentrations in bulk solution.^21^ These nanoclusters formed before macroscopic assemblies and promoted amyloid fibril formation upon aging, supporting the existence of finite intermediates that are not merely incomplete macroscopic condensates. Although these bulk nanoclusters are not equivalent to membrane-associated clusters, they provide experimental context for considering finite assemblies as distinct states along the αSyn assembly pathway. Recent cellular experiments further showed that lipid droplets promote condensate formation by wild-type and E46K αSyn. Lipid-droplet-rich condensates exhibited reduced fluorescence recovery and increased viscosity, yet their abundance and associated cellular dysfunction were substantially reversible when lipid-droplet availability was reduced.^22^ These observations indicate that lipid-rich interfaces can promote αSyn assembly and restrict condensate dynamics while retaining substantial reversibility. Within this context, it remains unclear what determines whether membrane-associated αSyn assemblies remain as finite or dynamically restricted surface states or instead acquire persistent amyloid-like intermolecular organization.^15,16,23^

One source of this state-selection ambiguity is that the distinct sequence regions of αSyn are functionally specialized but strongly coupled during membrane binding and self-assembly.^4,5,24^ The N-terminal region contains amphipathic repeats that mediate membrane recognition and membrane-induced helix formation;^3,4,25^ the central non-amyloid-β component (NAC) region provides aggregation-prone hydrophobic contacts required for amyloid assembly;^9^ and the acidic C-terminal region enhances solubility while contributing long-range electrostatic regulation.^4,9^ Membrane-bound αSyn can adopt broken, horseshoe-like, or extended helical conformations depending on lipid environment and experimental conditions.^26–28^ Membrane binding can therefore alter not only the spatial localization of αSyn but also the availability, orientation, and persistence of intra- and intermolecular contacts. Consistent with this view, cross-linking experiments have shown that transient N- and C-terminal interactions are reorganized upon membrane binding, indicating that αSyn coassembly with lipid interfaces involves contact-network remodeling rather than simple adsorption.^29^ A key challenge is to determine how N-terminal membrane anchoring and membrane-associated helical ordering reshape NAC contact organization associated with amyloid-like registry. Because membrane binding changes both the spatial localization and the conformational ensemble of αSyn, changes observed in membrane-associated assemblies cannot be attributed to interfacial recruitment alone. A controlled comparison between fully disordered and predefined membrane-bound-like helical/ordered representations in the absence of an explicit membrane is therefore required to isolate the effect of conformational ordering before the additional contribution of lipid-surface recruitment is introduced.

Beyond whether NAC contacts form, the relative orientation and registry of interacting NAC segments provide an additional structural distinction between prefibrillar and fibrillar states. ATR–FTIR measurements showed that toxic prefibrillar αSyn oligomers are enriched in antiparallel β-sheet organization, whereas mature fibrils adopt predominantly parallel in-register packing.^30^ More recently, atomic-resolution characterization of a lipid-induced toxic prefibrillar intermediate identified antiparallel β-strands that undergo structural reorganization during maturation into lipidic fibrils.^31^ These observations motivate examining both parallel-like and antiparallel-like NAC arrangements, because they may distinguish oligomer-like intermediate organization from fibril-like intermolecular registry.

These orientation- and registry-resolved NAC features therefore provide geometric criteria for assessing whether condensates acquire amyloid-like intermolecular organization. This distinction is important because condensation and fibrillization are related but nonequivalent processes. Liquid–liquid phase separation has been proposed as a potential noncanonical route for αSyn aggregation, in which liquid condensates concentrate aggregation-prone intermediates and may subsequently age toward amyloid-rich hydrogel or solid states.^32^ Consistent with this context dependence, αSyn liquid droplets have been observed to mature into amyloid-rich hydrogel-like assemblies, whereas model synaptic vesicles delayed this maturation process.^33^ αSyn condensates can increase local protein concentration and thereby promote amyloid nucleation or fibrillar growth under some conditions.^34,35^ However, condensates can suppress aggregation or remain dynamic without completing amyloid maturation,^36,37^ and their conversion toward amyloid states can be pharmacologically inhibited.^38^ More broadly, phase separation and fibrillization can be thermodynamically separable, indicating that condensate formation alone is insufficient to define amyloid maturation.^39^

Beyond condensate formation itself, reduced molecular mobility within a condensate should not automatically be interpreted as evidence of amyloid maturation. A kinetically restricted condensate may represent a step toward solidification or, alternatively, a frustrated surface-associated state in which global motion is slowed without necessarily stabilizing persistent parallel-like or antiparallel-like NAC registries. Distinguishing between these possibilities requires connecting condensate-scale dynamics with residue-level NAC contact persistence.

Here, we use coarse-grained multichain simulations to determine how an anionic POPC/POPG bilayer and membrane-bound-like helical ordering reshape αSyn condensates. By comparing fully disordered and predefined helical/ordered representations in solution and then at the membrane, we separate the effects of conformational ordering from lipid-surface recruitment and examine how membrane association alters condensate morphology, partitioning, dynamics, and NAC contact persistence. We find that predefined helical/ordered segments increase local condensate density, promote directional NAC packing, and prolong selected NAC contacts, yet weaken the global condensate cohesion at late simulation times. Membrane association anchors the N-terminal region, produces flattened surface-associated assemblies, and redistributes αSyn into populations outside the largest condensate. Although the membrane-associated condensates show reduced chain mobility and stronger subdiffusive behavior, they do not exhibit systematically longer registry-resolved NAC contact lifetimes. Together, these results indicate that membrane-driven surface assembly and kinetic restriction can be decoupled from persistent amyloid-like intermolecular organization.

## 2 Results

### 2.1 Predefined Helical Ordering Increases Local Condensate Density but Weakens Global Network Cohesion

The central question of this study is whether membrane-associated αSyn assembly reflects simple enrichment of protein at a lipid interface or a more selective remodeling of the condensate pathway. Before introducing an explicit membrane, we first isolated one membrane-associated structural effect: the ordered conformation adopted by the N-terminal/NAC-containing region in a membrane-bound-like state. We compared a fully disordered 50-chain system, denoted IDP_50_, with a predefined helical/ordered 50-chain system, denoted Hel_50_. In the IDP model, the entire 140-residue sequence was represented as intrinsically disordered. In the Hel model, residues 1–95 were retained in a membrane-associated helical/ordered representation derived from the micelle-bound αSyn structure 1XQ8,^25^ whereas residues 96–140 remained disordered (Figure 1A). This ordered region includes both the N-terminal membrane-binding repeats and the NAC segment. Thus, this soluble comparison separates membrane-associated conformational ordering from membrane-mediated interfacial recruitment and provides a baseline for determining how lipid-surface recruitment further reshapes αSyn assembly.

**Figure 1:**
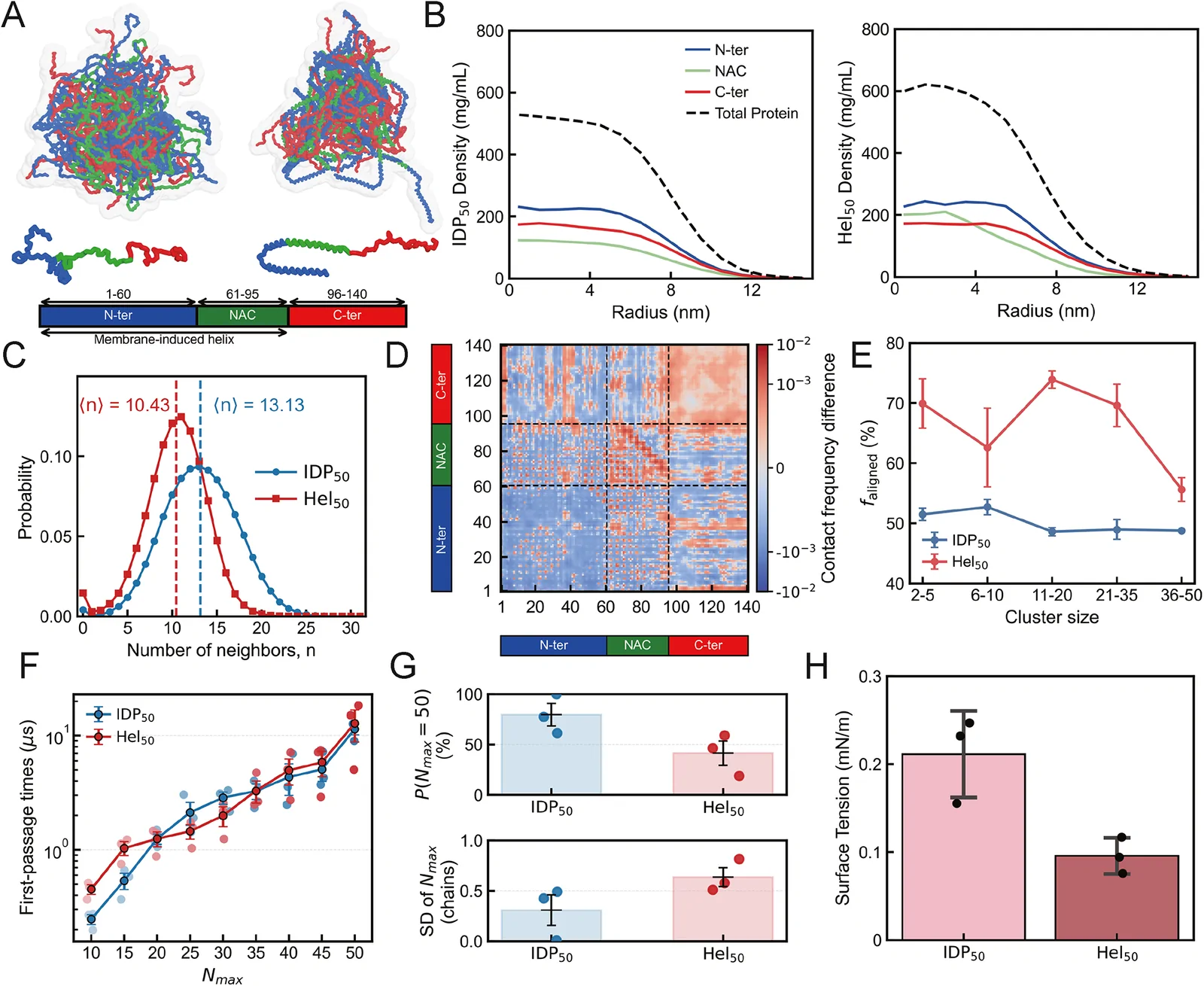
Predefined helical ordering increases local condensate density but weakens global αSyn condensate cohesion. (A) Representative condensate structures of IDP_50_ (left) and Hel_50_ (right), with the corresponding monomer representations shown below. The N-terminal, NAC, and C-terminal regions are colored blue, green, and red, respectively. (B) Segment-resolved radial mass-density profiles, showing higher core density in Hel_50_. (C) Interchain neighbor number distributions, showing higher chain-level coordination in IDP_50_. (D) Interchain residue contact-frequency difference map, calculated as Hel_50_–IDP_50_. Blue and red regions indicate contacts enriched in IDP_50_ and Hel_50_, respectively. (E) Percentage of directly NAC-contacting chain pairs with axial alignment, *f*_aligned_, defined by |cos θ| ≥ 0.5 for the N-to-C-oriented NAC PCA axes. (F) First-passage times for the largest cluster to reach selected *N*_max_ values. (G) Percentage of the final 10 μs spent in the fully condensed state, *N*_max_ = 50 (upper panel), and the SD of *N*_max_ over that interval (lower panel). (H) Apparent surface tension estimated from condensate shape fluctuations. Where shown, error bars indicate SD across three independent replicate trajectories.

Both IDP_50_ and Hel_50_ formed dense multichain assemblies from initially dispersed chains during the 20 μs simulations conducted in a 48.42 × 48.42 × 48.42 nm^3^ box at an effective protein concentration of ∼750 μM and 0.15 M NaCl (Figure 1A and Figure S1). Comparison of condensate properties during the final 10 μs revealed an apparent paradox. The Hel_50_ condensate had a higher core protein density than IDP_50_, with stronger contributions from the predefined N-terminal/NAC-containing region (Figure 1B). Nevertheless, this denser core did not correspond to higher chain-level coordination. The interchain neighbor distribution shifted toward larger coordination numbers in IDP_50_, with an average neighbor number of 13.13, whereas Hel_50_ had a lower average neighbor number of 10.43 (Figure 1C). These patterns are only weakly affected by reasonable variations in the elastic-network construction parameters, including the elastic-network cutoff and force constant, as well as by the intermolecular contact cutoff used in the analysis (Figures S2 and S3).

The interchain contact-frequency difference map further revealed how the sequence distribution of contacts differed between the two condensates. The map was calculated within condensates as Hel_50_ – IDP_50_, such that blue regions indicate contacts enriched in IDP_50_ and red regions indicate contacts enriched in Hel_50_ (Figure 1D and Figure S4). Consistent with the higher neighbor number of IDP_50_, the map was globally biased toward blue, showing stronger overall interchain contact connectivity in the fully disordered condensate. However, the Hel_50_ condensate retained selective local contact enrichment, particularly NAC antidiagonal-like contacts and contacts involving the C-terminal region. Therefore, the higher core density of Hel_50_ reflects localized and sequence-selective packing rather than global strengthening of the condensate contact-network. In contrast, the fully disordered chains support broader, more distributed interchain connectivity, providing a structural basis for their stronger global condensate cohesion.

We next asked whether the local contact features enriched in Hel_50_ were associated with directional NAC packing. For each directly NAC-contacting chain pair, we calculated the signed relative orientation, cos θ, between the N-to-C-oriented PCA axes of the two NAC segments and classified a pair as axially aligned when |cos θ| ≥ 0.5. Hel_50_ showed a higher percentage of axially aligned NAC-contacting pairs than IDP_50_ across all cluster-size regimes, with the clearest difference in the 11–35-chain intermediate regime; the difference was smaller for large clusters containing 36–50 chains (Figure 1E). Thus, predefined helical/ordered segments promote axial NAC organization most clearly in the intermediate-cluster regime. The signed cos θ distribution further showed that this axial organization includes both parallel-like and antiparallel-like configurations rather than a single dominant antiparallel population (Figure S5A). A broad NAC antidiagonal contact enrichment was also positive on average in intermediate-sized Hel_50_ clusters, although this registry-level signal was more variable across replicates (Figure S5B). These results support enhanced directional NAC packing in Hel_50_, while avoiding a stronger interpretation that the condensate is dominated by a purely antiparallel architecture. The inclusion of antiparallel-like and antidiagonal metrics was motivated by experimental evidence that toxic prefibrillar αSyn oligomers can contain antiparallel β-sheet organization, whereas mature fibrils are dominated by parallel in-register packing.^30,31^ The transient antidiagonal enrichment observed in intermediate-sized Hel_50_ clusters is therefore consistent with an oligomer-like head-to-tail topological tendency, but it should not be interpreted as direct evidence of antiparallel β-sheet formation.

This structural picture explains why the kinetic effect of predefined helical/ordered segments was selective rather than global. First-passage analysis revealed a cluster-size-dependent crossover in assembly kinetics. IDP_50_ was faster in the small-cluster regime, whereas Hel_50_ reached intermediate-sized clusters more rapidly, particularly for 20 ≤ *N*_max_ ≤ 35, and IDP_50_ regained the kinetic advantage as the system approached the fully condensed state (Figure 1F). Predefined helical/ordered segments therefore do not simply accelerate early aggregation. Rather, they bias the assembly pathway toward an intermediate-cluster regime, consistent with directional local packing that favors intermediate-cluster capture over unrestricted condensate coarsening.

The intermediate-cluster advantage of Hel_50_ did not translate into greater persistence of the fully condensed state. During the last 10 μs, IDP_50_ occupied the fully condensed state, *N*_max_ = 50, for 79.6% of the sampled time, whereas Hel_50_ did so for only 41.5% (Figure 1G). The fluctuations of *N*_max_ were also larger in Hel_50_, indicating reduced persistence of the maximally condensed state. Consistently, condensate shape-fluctuation analysis yielded a higher apparent surface tension for IDP_50_ than for Hel_50_ (Figure 1H), supporting stronger global dense-phase cohesion in the fully disordered system.

Together, these results show that predefined membrane-associated helical/ordered segments do not act as a general stabilizer of αSyn condensation. Instead, they generate a locally dense and directionally packed condensate state that selectively favors intermediate-cluster capture but limits the multivalent reorganization required for robust condensate cohesion. This membrane-free baseline distinguishes conformational ordering from interfacial recruitment and provides the reference needed to determine whether an anionic membrane stabilizes condensates directly or redirects αSyn into surface-associated cluster states.

### 2.2 Predefined Helical Ordering Strengthens N-Terminal Membrane Anchoring without Strongly Reducing Lateral Mobility

The soluble comparison above showed that membrane-bound-like ordering is not equivalent to stronger global condensate cohesion. We next asked how the same predefined helical/ordered representation affects direct interaction with an anionic membrane. This single-chain membrane analysis was used as a control for the multichain membrane systems below: it tests whether the helical/ordered model strengthens membrane anchoring and whether such anchoring by itself substantially changes lateral mobility on the bilayer surface.

We constructed two single-chain membrane systems, denoted IDP_1_-Mem and Hel_1_-Mem, above a POPC/POPG = 7:3 bilayer (Figure 2A). During the last 5 μs, Hel_1_-Mem remained closer to the bilayer than IDP_1_-Mem, as reflected by a smaller membrane-normal center-of-mass distance between the protein and the membrane (Figure 2B and Figure S6). Residue-wise protein–membrane contact analysis further showed that this enhanced binding was concentrated mainly in the N-terminal region (Figure 2C and Figure S7), consistent with the amphipathic membrane-binding role of the N-terminal repeats.^3^ Thus, predefined helical/ordered segments increase the stability of single-chain membrane association primarily by strengthening N-terminal membrane anchoring.

**Figure 2:**
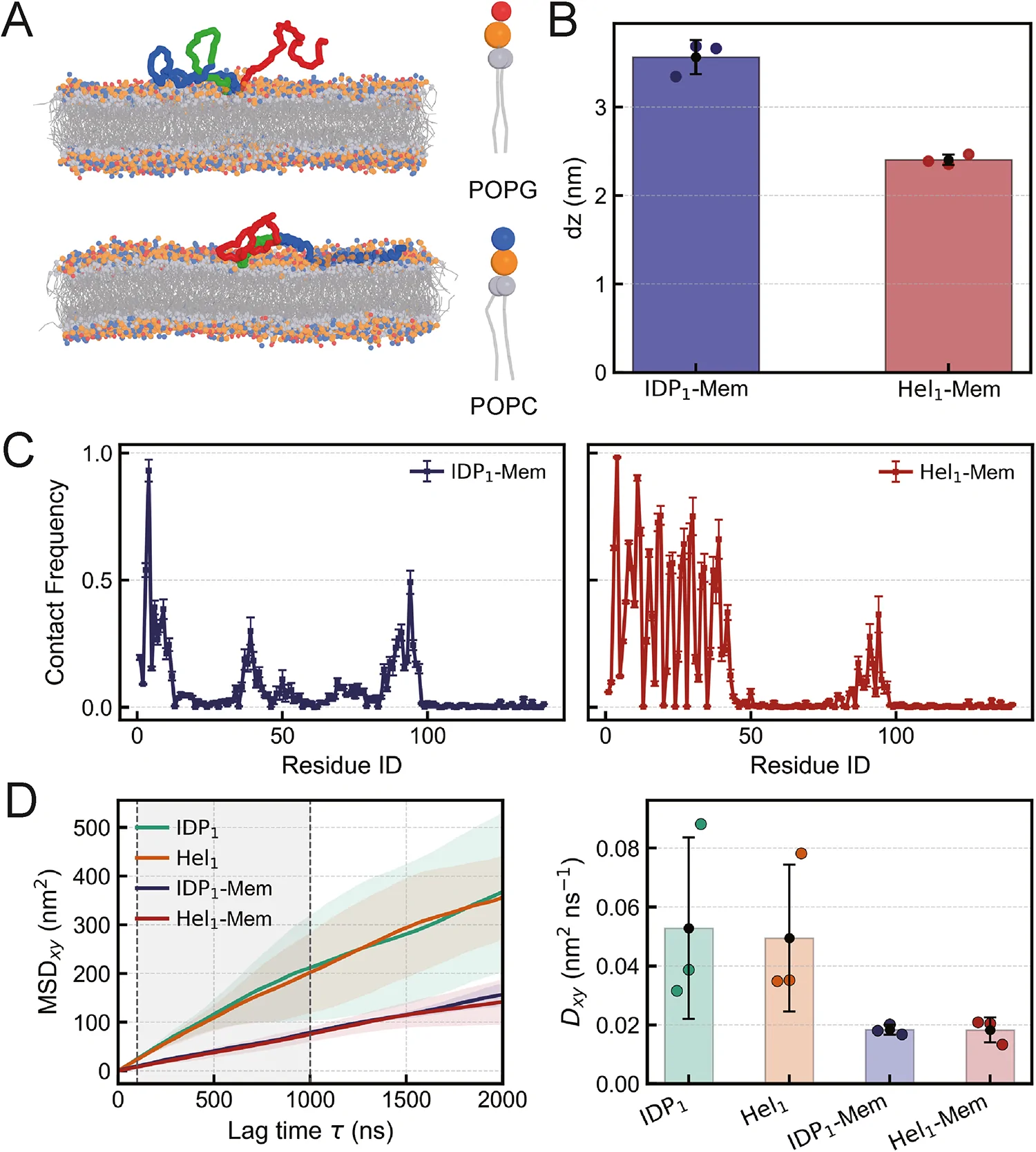
Predefined helical ordering strengthens single-chain membrane anchoring without strongly reducing lateral mobility. (A) Representative structures of IDP_1_-Mem (top) and Hel_1_-Mem (bottom) on a POPC/POPG = 7:3 bilayer. The N-terminal, NAC, and C-terminal regions are colored blue, green, and red, respectively. (B) Membrane-normal protein–membrane center-of-mass distance, showing closer membrane association for Hel_1_-Mem. (C) Residue-wise membrane-contact-frequency, showing enhanced N-terminal membrane contacts in Hel_1_-Mem. (D) In-plane (*xy*) mean-squared displacement (left) and fitted lateral diffusion coefficient, *D*_*xy*_ (right), for the two soluble and two membrane-associated monomer systems. The membrane-associated monomers diffuse more slowly than their corresponding soluble controls, whereas IDP_1_-Mem and Hel_1_-Mem show comparable lateral mobility. Where shown, error bars indicate SD across three independent replicate trajectories.

Despite the stronger anchoring of Hel_1_-Mem, the two states showed similar lateral mobility. Their MSD_*xy*_ curves and fitted *D*_*xy*_ values were comparable (Figure 2D). In contrast, both membrane-bound monomer states diffused more slowly in the membrane plane than their corresponding soluble monomer states. These results indicate that the POPC/POPG bilayer imposes a general membrane-plane mobility constraint, whereas predefined helical/ordered segments mainly enhance membrane anchoring without strongly reducing single-chain lateral diffusion. This distinction is important for interpreting the multichain systems: any additional slowdown observed in helical-segment-containing membrane condensates cannot be attributed simply to slower diffusion of an isolated helical chain on the membrane.

### 2.3 Anionic Membranes Support Surface-Associated Condensates with N-Terminal Membrane Anchoring and Weakened Largest-Condensate Partitioning

The single-chain analysis showed that predefined helical/ordered segments strengthen membrane anchoring. In contrast, the soluble 50-chain comparison revealed that membrane-bound-like ordering alone weakens global condensate cohesion. We therefore next asked how membrane association and predefined helical ordering combine in multichain assemblies. To focus on membrane-induced remodeling of condensate architecture and partitioning, rather than on concentration-dependent condensate formation, the membrane simulations were performed at the same effective protein concentration as the soluble 50-chain systems, where bulk condensates form reproducibly from initially dispersed chains. We compared a membrane-associated fully disordered system, IDP_50_-Mem, with a mixed system, Hel_10_IDP_40_-Mem, containing 10 predefined helical/ordered chains and 40 fully disordered chains above a POPC/POPG = 7:3 bilayer (Figure S8; see Section 4 for details). In the analyzed membrane-associated trajectories, both systems contained large assemblies localized at the membrane interface (Figure 3A). Compared with the soluble condensates, the membrane-associated assemblies were visibly flattened along the membrane-normal direction and spread laterally over the bilayer surface. This morphology indicates that membrane association reorganizes αSyn condensates into interfacial assemblies rather than bulk-like spherical droplets. In both IDP_50_-Mem and Hel_10_IDP_40_-Mem, the largest assemblies remained membrane associated rather than forming bulk-like spherical condensates.

**Figure 3:**
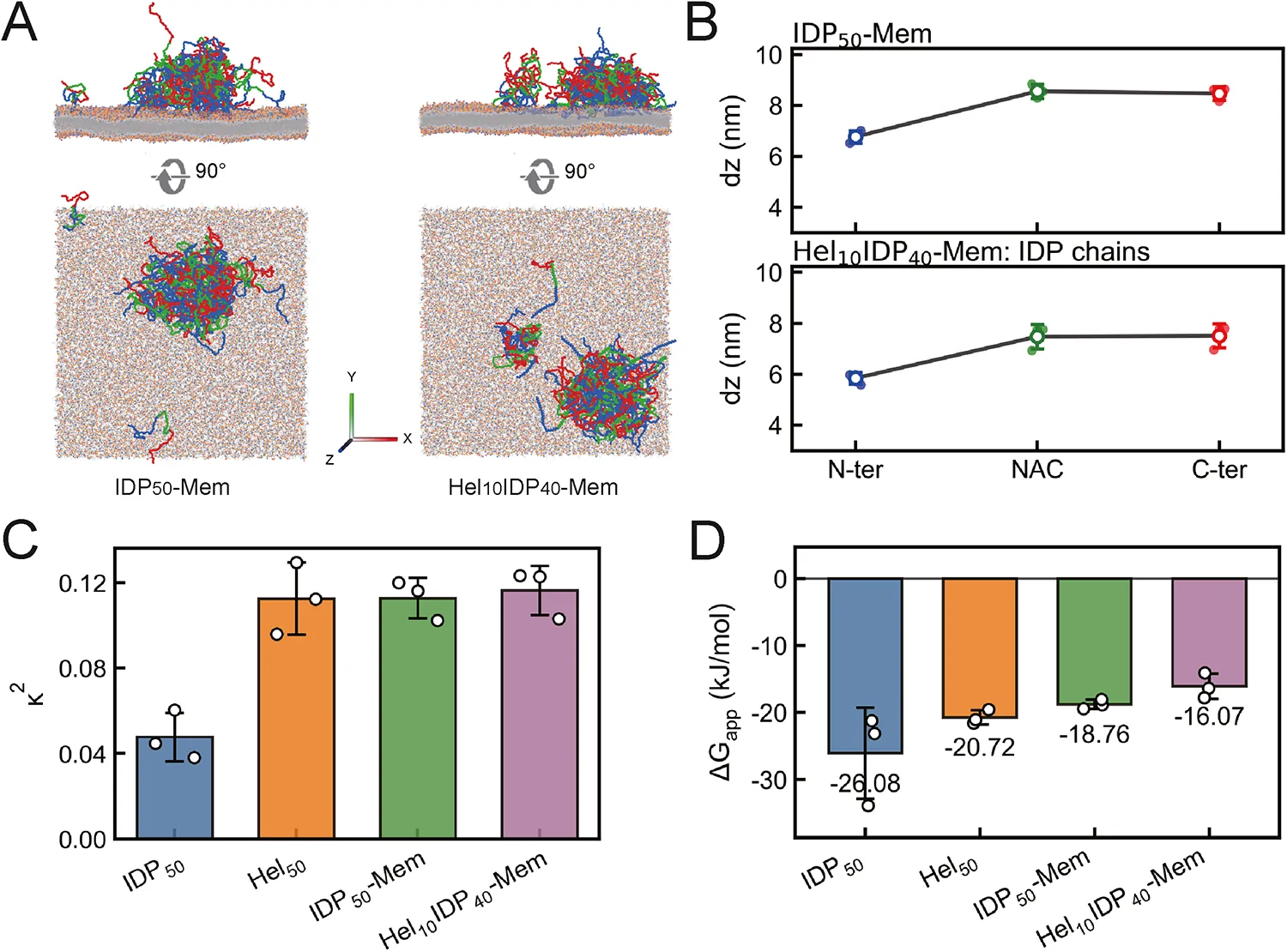
Anionic membranes support flattened, N-terminal-anchored surface-associated αSyn condensates with weakened apparent partitioning into the largest condensate. (A) Representative side and top views of IDP_50_-Mem and Hel_10_IDP_40_-Mem assemblies on a POPC/POPG = 7:3 bilayer. The N-terminal, NAC, and C-terminal regions are colored blue, green, and red, respectively. (B) Segment-resolved membrane-normal positions in the membrane-associated assemblies. The membrane-normal center-of-mass displacement relative to the membrane midplane, *d*_*z*_, was calculated for the N-terminal, NAC, and C-terminal regions in IDP_50_-Mem and for the fully disordered chains in Hel_10_IDP_40_-Mem. (C) Shape anisotropy, κ^2^, of soluble and membrane-associated condensates. Higher κ^2^ values indicate stronger deviation from an isotropic droplet-like morphology. (D) Density-derived apparent transfer free energy, Δ*G*_app_ = −*RT *ln­(ρ_dense_/ρ_dilute_), calculated from the fitted dense- and dilute-phase plateau densities. Less negative Δ*G*_app_ values indicate weakened apparent partitioning into the largest condensate in systems with predefined helical/ordered segments and membrane association. Density profiles were fitted independently for each replicate trajectory; bars and error bars indicate the mean and SD of the three replicate-wise Δ*G*_app_ estimates.

For IDP_50_-Mem and the fully disordered chains in Hel_10_IDP_40_-Mem, the segment-resolved membrane-normal distributions showed that this interfacial organization was associated with preferential N-terminal localization near the bilayer. During the last 10 μs, the N-terminal segments were enriched closest to the membrane surface, whereas the NAC and C-terminal regions were distributed farther from the bilayer and over a broader range of membrane-normal positions (Figure 3B and Figure S9). Thus, the N-terminal membrane-binding preference observed in the single-chain systems is preserved within multichain surface condensates. This anchoring geometry provides a structural basis for the flattened morphology: the bilayer recruits the N-terminal segments to the interface while the remaining regions extend away from the surface and participate in protein–protein contacts.

To quantify the nonspherical character of these assemblies, we calculated the shape anisotropy parameter, κ^2^, for the largest condensate (Figure 3C and Figure S10). The soluble IDP_50_ condensate showed the lowest κ^2^, consistent with a more isotropic droplet-like morphology. In contrast, Hel_50_, IDP_50_-Mem, and Hel_10_IDP_40_-Mem all showed higher κ^2^ values, indicating stronger deviation from an isotropic spherical shape. Because κ^2^ reports shape anisotropy rather than the direction of deformation, this metric alone does not define the assemblies as flattened. However, together with the side and top views in Figure 3A, the elevated κ^2^ values support the interpretation that membrane-associated condensates adopt flattened, surface-associated architectures rather than bulk-like spherical morphologies.

We then quantified how membrane association changed the partitioning of αSyn into the largest condensate using radial density profiles and a density-derived apparent transfer free energy, Δ*G*_app_ = −*RT* ln­(ρ_dense_/ρ_dilute_) (Figure 3D and Figure S11), where ρ_dense_ and ρ_dilute_ are the dense- and dilute-phase plateau densities obtained from the hyperbolic-tangent fit. Among the soluble systems, IDP_50_ showed the most favorable apparent partitioning into the largest condensate, whereas Hel_50_ was less favorable despite its higher fitted dense-phase density, because its fitted dilute-phase density was also higher, reflecting a larger protein population outside the largest condensate. Membrane association further reduced the apparent condensation tendency: IDP_50_-Mem and Hel_10_IDP_40_-Mem both showed less negative Δ*G*_app_ values than the soluble condensates, with the mixed membrane-associated system showing the weakest partitioning into the largest condensate. The stability of these density-based results was further supported by the close agreement of radial density profiles calculated over progressively extended 5–10 μs analysis windows (Figure S12).

Importantly, these changes were driven primarily by an increase in the fitted dilute-phase density, ρ_dilute_, rather than by a collapse of the fitted dense-phase density, ρ_dense_ (Figure S13). The fitted dense-phase densities remained broadly comparable among systems, whereas the fitted dilute-phase densities increased markedly in the presence of predefined helical/ordered segments and membrane association. Therefore, Δ*G*_app_ is interpreted here as an apparent partitioning metric rather than a rigorous phase-coexistence free energy. Under this interpretation, the membrane does not simply stabilize or destabilize a bulk-like condensate. Instead, it redistributes αSyn among the largest surface-associated condensate, smaller membrane-associated populations, and the surrounding dilute-phase.

### 2.4 Membrane Association Restricts Condensate Dynamics without Systematically Prolonging NAC Contact Persistence

Membrane association substantially reshapes condensate morphology and partitioning, but whether it promotes persistent NAC contact organization requires a separate test. A slower condensate is not necessarily a more amyloid-competent condensate: kinetic restriction at the condensate-scale must be distinguished from stabilization of aggregation-prone NAC contacts. We therefore compared condensate dynamics and NAC contact persistence in the soluble IDP_50_ condensate, the fully disordered membrane condensate IDP_50_-Mem, and the mixed membrane-associated condensate Hel_10_IDP_40_-Mem. The soluble Hel_50_ condensate was included as an ordered but membrane-free control to distinguish the effects of predefined helical/ordered segments from those arising from membrane association.

We first calculated the three-dimensional MSD of chains retained in the largest condensate during the last 10 μs (Figure 4A). Membrane-associated condensates showed substantially reduced MSD compared with the soluble IDP_50_ condensate, indicating that the membrane interface restricts collective chain mobility within the dense-phase. The soluble Hel_50_ condensate showed an intermediate reduction in MSD relative to IDP_50_, whereas Hel_10_IDP_40_-Mem showed the strongest slowdown. Because membrane-associated assemblies can have anisotropic motion, we also calculated the membrane-plane MSD, MSD_*xy*_. The same trend was observed in MSD_*xy*_, showing that the slowdown is not simply an artifact of restricting membrane-normal motion. Together with the single-chain diffusion analysis (Figure 2D), these results indicate that the additional slowdown in Hel_10_IDP_40_-Mem is a condensate-scale property rather than a direct consequence of slower isolated-chain diffusion on the membrane.

**Figure 4:**
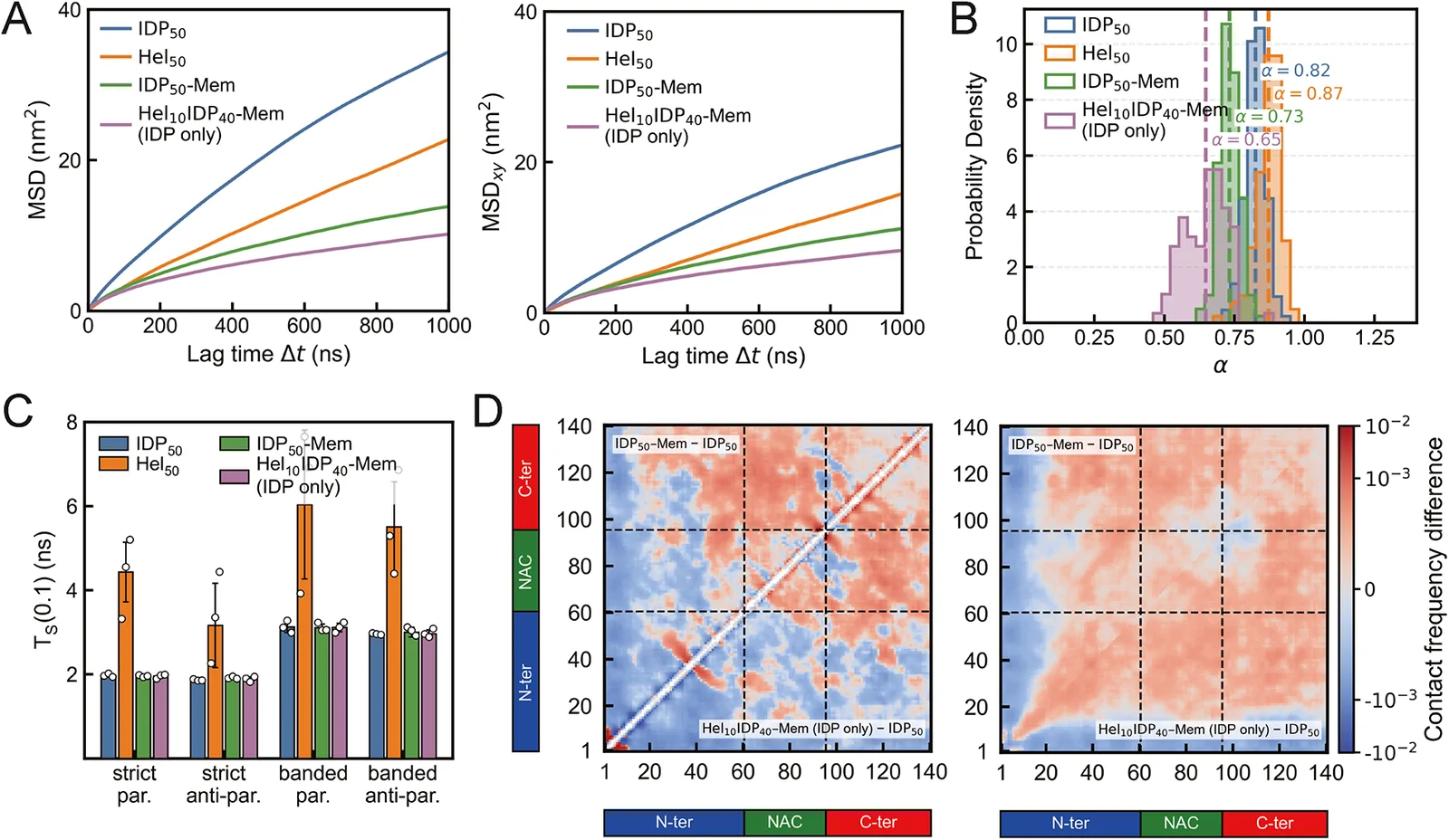
Membrane association restricts condensate dynamics without systematically prolonging persistent NAC contacts. (A) Three-dimensional MSD and membrane-plane MSD_*xy*_ of chains retained in the largest condensate during the last 10 μs. The soluble Hel_50_ condensate is included as an ordered but membrane-free control. (B) Probability-density distributions of the anomalous diffusion exponent α, obtained from MSD­(τ) ∼ τ^α^. Dashed vertical lines and numerical labels indicate the mean α values. (C) Characteristic continuous NAC contact-survival times, T_S_(0.1), defined as the first time at which the empirical survival probability decreases to 0.1. The four criteria require at least three consecutive strict parallel-like contacts (strict par.), strict antiparallel-like contacts (strict antipar.), banded parallel-like contacts (banded par.), or banded antiparallel-like contacts (banded antipar.). Longer consecutively ordered contact segments also satisfy these criteria. The strict criteria require exact forward or reverse sequence correspondence, whereas the banded criteria permit a limited registry displacement while preserving forward or reverse ordering. For Hel_10_IDP_40_-Mem, panel (C) reports contacts between the fully disordered chains only. Bars indicate means and error bars indicate SD across three independent trajectories. (D) Intrachain (left) and interchain (right) residue–residue contact-frequency difference maps relative to IDP_50_. In each map, the upper triangle shows IDP_50_-Mem minus IDP_50_, whereas the lower triangle shows Hel_10_IDP_40_-Mem minus IDP_50_, calculated using only the fully disordered chains in the mixed membrane system. Dashed lines mark the boundaries of the N-terminal, NAC, and C-terminal regions.

To quantify deviations from normal diffusion, we fitted MSD­(τ) ∼ τ^α^ and extracted the anomalous diffusion exponent α (Figure 4B and Figure S14). The membrane-associated condensates exhibited lower α values than the soluble condensates, with mean values decreasing from 0.82 in IDP_50_ and 0.87 in Hel_50_ to 0.73 in IDP_50_-Mem and 0.65 in Hel_10_IDP_40_-Mem. Thus, although predefined helical/ordered segments reduced the magnitude of chain displacement in the soluble Hel_50_ condensate, they did not independently reproduce the stronger subdiffusive behavior observed in the membrane-associated systems. However, even the slowest system retained α > 0.6, indicating that the condensates remained dynamically mobile rather than kinetically frozen. Reduced molecular mobility and enhanced subdiffusive behavior are often associated with condensate aging and progression toward amyloid formation. Whether the dynamical restriction observed here reflects such structural maturation, however, cannot be determined from translational dynamics alone. We therefore next examined whether the membrane-induced slowdown was accompanied by prolonged intermolecular NAC contacts.

Continuous interchain NAC contact episodes were quantified using four definitions requiring ≥3 consecutively ordered NAC residues (Figure 4C). For Hel_10_IDP_40_-Mem, the analysis in Figure 4C was restricted to contacts between the fully disordered chains (IDP–IDP pairs). The strict parallel-like criterion required at least three consecutive residues on one NAC segment to contact the same sequence indices on the partner segment, representing an exact same-register arrangement (strict par.). The strict antiparallel-like criterion required at least three consecutive residues to contact the corresponding partner residues in reverse sequence order (strict antipar.). We additionally introduced banded parallel-like and banded antiparallel-like criteria to account for locally shifted registries. These banded definitions preserved the forward or reverse consecutive ordering of the contacting residues but permitted a limited displacement from the exact diagonal (banded par.) or antidiagonal (banded antipar.) registry. For each definition, a continuous episode ended as soon as the complete ≥ 3-consecutive-residue criterion was no longer satisfied. The long-lived portion of the contact-duration distribution was summarized by T_S_(0.1), defined as the first time at which the empirical survival probability decreased to 0.1.

Despite their substantially lower MSD and anomalous diffusion exponents, neither IDP_50_-Mem nor the IDP–IDP pairs in Hel_10_IDP_40_-Mem showed a systematic increase in T_S_(0.1) relative to soluble IDP_50_ (Figure 4C). This conclusion held for the strict parallel-like and strict antiparallel-like three-consecutive-residue criteria and for their more permissive banded parallel-like and banded antiparallel-like counterparts. Allowing a limited registry displacement increased the absolute survival times, but it did not reveal preferential stabilization of NAC contacts satisfying these consecutive-residue criteria in the membrane-associated condensates. The membrane-induced reduction in chain mobility therefore did not translate into a corresponding increase in the uninterrupted residence of registry-resolved NAC contacts.

By comparison, the soluble Hel_50_ control showed longer contact-survival times under all four three-consecutive-residue criteria, with the largest differences observed for the banded criteria. This result demonstrates that the contact-survival analysis is sensitive to local stabilization produced by predefined structural ordering. However, these longer-lived NAC contacts coexist with the lower average neighbor number, weaker global connectivity, and reduced persistence of the fully condensed state identified for Hel_50_ above. The soluble Hel_50_ control therefore establishes that stabilization of selected local NAC arrangements is not by itself sufficient to strengthen the global condensate network. Together with the membrane-associated systems, which exhibited condensate-scale dynamical restriction without prolonged NAC contact persistence, these results show that local NAC contact stabilization and global condensate dynamics can vary independently. This conclusion was robust across the broader hierarchy of NAC contact definitions, registry windows of 4–10 residues, pairwise Kolmogorov–Smirnov comparisons of the complete contact-lifetime distributions, and the complementary contact-state autocorrelation analysis (Figures S15–S20). Across these analyses, Hel_50_ consistently exhibited prolonged NAC contact persistence, whereas IDP_50_-Mem and the IDP–IDP pairs in Hel_10_IDP_40_-Mem remained broadly comparable to IDP_50_. Pair-type-resolved analysis of Hel_10_IDP_40_-Mem further showed generally longer-lived Hel–Hel contacts than IDP–IDP contacts, with Hel–IDP pairs showing intermediate behavior in most cases (Figure S21).

Residue-level contact-frequency maps provided a structural context for this dynamical decoupling (Figure 4D). Intrachain difference maps showed reduced N-terminal contacts and localized increases in C-terminal contacts upon membrane association, consistent with engagement of the N-terminal region by the bilayer. Interchain maps likewise showed reduced N-terminal-mediated contacts together with localized redistribution of NAC- and C-terminal interactions. For Hel_10_IDP_40_-Mem, the difference maps were calculated using only the fully disordered chains, demonstrating that contact-network remodeling also extends to chains that were not directly subjected to the membrane-localizing restraint. Importantly, these frequency changes represent redistribution of the accessible contact-network rather than stabilization of continuously persistent NAC registry.

Together, these results distinguish three organizational properties that need not vary together: condensate-scale mobility, global intermolecular network cohesion, and residue-level NAC contact persistence. Membrane association produces surface-associated condensates with reduced chain mobility and stronger subdiffusive behavior, but it does not systematically prolong exact or locally shifted NAC contact registries in either parallel-like or antiparallel-like orientations. The soluble Hel_50_ control further shows that longer-lived local NAC contacts can occur without stronger global condensate cohesion. The membrane-associated assemblies are therefore best described as kinetically restricted and contact-reorganized surface condensates rather than condensates with broadly stabilized persistent NAC organization.

## 3 Discussion and Conclusions

Recent studies have produced apparently divergent views of membrane-associated αSyn assembly. On the one hand, membrane interfacial potentials can recruit αSyn at concentrations below the bulk saturation threshold, promote surface condensation, and provide nucleation sites for subsequent fibril formation.^16^ On the other hand, membrane-bound αSyn has also been reported to undergo self-limiting multimerization, producing finite-sized and dynamically exchanging assemblies rather than continuous growth into macroscopic condensates.^15^ Our results suggest that these observations need not represent mutually exclusive outcomes. Instead, membrane-driven surface assembly and condensate-scale kinetic restriction can be decoupled from global condensate cohesion and persistent amyloid-like intermolecular organization. An anionic membrane may therefore promote the accumulation and local organization of αSyn without necessarily directing all recruited proteins toward a single, continuously maturing assembly state.

The soluble systems clarify this separation: predefined ordering based on the membrane-bound conformation increased local protein density and promoted directional NAC packing, particularly during the formation of intermediate-sized clusters. However, these locally organized contacts did not produce a more extensively connected condensate network or a more persistent fully condensed state. A related region-specific structure–dynamics decoupling has recently been reported for TDP-43 condensates, in which helical regions formed stronger but shorter-lived interaction hubs, whereas intrinsically disordered regions established weaker yet more persistent contacts.^40^ Together with our results, this comparison emphasizes that local contact strength, contact persistence, and global condensate cohesion need not vary in parallel. One interpretation is that conformational ordering redistributes effective interaction valency rather than uniformly increasing it. Restricting part of the chain to a limited set of conformations and relative orientations can favor locally compatible packing geometries, while reducing the large number of alternative contact configurations accessible to fully disordered chains. The resulting assemblies can therefore be locally dense but globally less cohesive. In this view, disorder supports condensate cohesion not simply by exposing attractive residues, but by providing the configurational adaptability required to construct and continuously reorganize a distributed multivalent interaction network.^41,42^ Predefined ordering instead concentrates association into a smaller subset of geometrically favorable contacts, promoting cluster capture while limiting the collective rearrangements required for unrestricted coarsening.

Membrane recruitment further shifts this balance by coupling protein–membrane binding to protein–protein assembly. N-terminal anchoring increases the local population of αSyn at the bilayer and confines the proteins to an anisotropic interfacial environment. This recruitment can raise surface occupancy even when a larger fraction of the proteins remains outside the largest condensate. The membrane should therefore not be regarded simply as a catalyst that increases the effective concentration of an otherwise unchanged bulk condensate. It creates a distinct ensemble of interfacial states, including membrane-bound monomers, finite surface clusters, and larger flattened assemblies, whose relative populations depend on the competition between protein–membrane and protein–protein interactions. This interpretation is consistent with recent coarse-grained simulations and experiments showing that negatively charged lipids can initially enhance membrane recruitment but, when protein–lipid interactions become sufficiently strong, compete with protein–protein interactions and disperse membrane-associated condensates.^43^ Thus, increased surface occupancy need not imply stronger partitioning into a single cohesive condensate. Stronger membrane association may increase recruitment while simultaneously restricting the orientations and configurational rearrangements needed to construct a globally cohesive intermolecular network. This competition provides a physical explanation for why membrane-associated αSyn can accumulate into prominent surface assemblies without all recruited molecules being incorporated into the largest condensate.

The dynamical results reveal a related distinction between kinetic restriction and molecular contact stabilization. Membrane-associated condensates displayed lower chain mobility and stronger subdiffusive behavior, but these changes were not accompanied by slower relaxation across the registry-resolved NAC contact classes monitored here, including strict and banded parallel-like and antiparallel-like definitions. A reduction in condensate-scale mobility is therefore not sufficient evidence that the aggregation-prone regions have developed a more persistent amyloid-associated NAC registry, whether parallel in-register-like or antiparallel/head-to-tail-like. Membrane anchoring, geometric confinement, crowding, and collective surface motion can all reduce translational mobility without stabilizing specific intermolecular contact patterns. This distinction is relevant to the interpretation of experimental mobility measurements: reduced diffusion or slower fluorescence recovery may indicate a more constrained assembly, but neither measurement alone establishes progression toward amyloid-like organization. This interpretation is consistent with recent cellular measurements showing that lipid-droplet-rich αSyn condensates exhibit reduced fluorescence recovery and increased viscosity while retaining substantial reversibility upon lipid-droplet depletion.^22^ Although a lipid-droplet monolayer differs from the planar phospholipid bilayer considered here, both systems show that lipid association can jointly promote condensate formation and kinetic restriction without mobility measurements alone specifying the underlying intermolecular registry. More broadly, high mesoscopic viscosity can coexist with rapid local molecular rearrangements inside biomolecular condensates, further illustrating that condensate-scale material behavior and residue-level interaction dynamics are distinct observables.^44^ Evaluating amyloid-like organization requires combining condensate-scale dynamics with residue-specific or registry-specific measurements. In the present systems, membrane association produces kinetic restriction and contact-network remodeling, but does not broadly stabilize the parallel-like or antiparallel-like NAC registry classes used here as geometric proxies for amyloid-associated intermolecular alignment.

The separation among surface assembly, kinetic restriction, and persistent amyloid-like intermolecular organization may extend beyond αSyn. Lipid interfaces have been shown to promote both condensation and interfacial ordering of the FUS low-complexity domain, demonstrating that lipid recruitment can reshape not only local protein concentration but also the structural organization of an IDP-rich phase.^45^ In the membrane-associated LAT-Grb2-SOS system, limited availability of multivalent bonds can kinetically frustrate condensation and generate dynamically arrested, noncompact assemblies, illustrating that two-dimensional recruitment does not necessarily lead to unrestricted coarsening.^46^ Synapsin provides a physiological example in which a protein-rich liquid phase recruits and organizes lipid vesicles without implying pathological solidification.^47^ Together with the present results, these systems suggest that surface recruitment, finite cluster formation, kinetic restriction, global condensate cohesion, and persistent amyloid-like intermolecular organization should be treated as distinct observables rather than as interchangeable signatures of a single phase separation pathway.

Condensate-membrane coupling is also bidirectional. Whereas the present study focuses primarily on how a bilayer remodels αSyn assembly, membrane-associated protein condensates can in turn reorganize lipid distributions and alter membrane material properties.^48^ LAT-associated condensates, for example, can couple to and stabilize ordered lipid domains, demonstrating reciprocal communication between protein condensation and membrane organization.^49^ Membrane-associated αSyn condensation and fibrillation have likewise been linked to increased membrane rigidity, deformation, and eventual rupture.^16,50^ The explicit bilayer representation used here provides a framework for extending the analysis in this direction. Future simulations could determine whether αSyn-rich surface assemblies induce local lipid sorting, change lipid lateral diffusion, alter membrane thickness or acyl-chain order, generate curvature, or facilitate water and ion penetration. Such analyses would help establish whether the membrane-associated state identified here is mechanically benign, functionally adaptive, or capable of evolving into a membrane-damaging assembly.

A limitation of the present study is that the membrane is represented by a simplified planar POPC/POPG bilayer rather than by the compositional and structural heterogeneity of neuronal membranes; leaflet asymmetry, curvature, cholesterol-dependent packing, and heterogeneous lipid-packing defects are therefore not explicitly represented. Such membrane properties can influence not only αSyn adsorption but also its interfacial assembly and aggregation pathway.^51,52^ For example, Shaikh et al. showed that a specific PC:PS composition promotes surface condensation of αSyn at nanomolar concentrations and subsequent fibril nucleation, with numerical modeling describing this behavior as a membrane-induced prewetting-like transition.^16^ Lavasani et al. further showed that reducing anionic-lipid content weakens NAC region membrane sequestration and increases its solvent exposure, thereby favoring intermolecular association in a lipid-to-protein ratio-dependent manner,^53^ while high cholesterol content can substantially accelerate αSyn aggregation.^54^ Lipid-packing defects and lipid-to-protein ratio likewise regulate membrane-bound geometry and the balance between stable membrane association and amyloid formation.^55,56^ These observations support our view that membrane composition can shift the competition between surface enrichment and intermolecular network formation, thereby altering the balance among dispersed membrane-bound chains, finite clusters, condensates, and amyloid-prone assemblies. Future extensions could incorporate more realistic neuronal-like membrane compositions, curvature and asymmetry, together with protein interactions capable of stabilizing persistent cross-β/amyloid organization, to directly test how membrane composition controls progression from surface clustering to fibril formation.^57^

The molecular representation used in this work provides both an important advantage and a clear boundary for these conclusions. Many efficient residue-level models developed for IDP conformational ensembles and phase behavior, including HPS-type and MOFF-family models, treat solvent effects implicitly and are optimized primarily against single-chain dimensions, folded-state stability, or condensate properties.^58–60^ Martini 3-IDP instead retains explicit coarse-grained water, ions, lipids, and proteins within a common interaction framework, enabling solvent exposure, protein–membrane association, and multichain condensation to be examined simultaneously.^61^ This molecular scope comes with substantially greater computational cost than one-bead-per-residue implicit-solvent models. The present simulations, which approach the million-particle scale and extend to 20 μs with independent replicates, therefore provide extensive sampling of membrane-coupled multichain organization within the selected coarse-grained representation. This improves the robustness of comparisons among the modeled states, but does not eliminate uncertainties associated with the force field or imposed secondary-structure representation.

Importantly, the standard coarse-grained representations considered here are not designed to resolve spontaneous conversion from disordered or helical conformations into a cross-β amyloid architecture. The persistence of registry-resolved NAC contacts was therefore used as an indirect structural indicator of whether membrane association stabilizes amyloid-associated intermolecular organization, with parallel-like definitions representing fibril-like in-register alignment and antiparallel-like definitions representing head-to-tail arrangements relevant to prefibrillar organization, rather than as a direct measurement of fibril formation. The absence of prolonged relaxation across these contact classes argues against enhanced stabilization of the monitored registry-resolved NAC arrangements, but does not exclude slower changes in other NAC geometries or a subsequent secondary-structure transition outside the accessible model ensemble and simulation time scale. Models that combine condensate formation with dynamic secondary-structure conversion, anisotropic backbone hydrogen bonding, and explicit β-sheet registry would make it possible to quantify the membrane-dependent liquid-to-solid transition more directly. Such developments would help determine whether the kinetically restricted surface assemblies observed here are off-pathway states, long-lived intermediates, or precursors that require additional changes in membrane composition or protein conformation before fibril formation.

These considerations lead to several testable predictions. Increasing membrane affinity should not necessarily produce a monotonic increase in condensate cohesion: stronger recruitment may first enhance surface clustering but subsequently trap proteins in membrane-bound populations that are poorly incorporated into the largest condensate. Similarly, conditions that reduce αSyn mobility should not necessarily increase the lifetime of registry-resolved NAC contacts, including parallel-like or antiparallel-like arrangements, or fibril-seeding activity. Finally, changes in membrane charge, cholesterol content, packing defects, or curvature may shift the balance among finite surface clusters, extended interfacial condensates, and assemblies capable of developing persistent amyloid-like intermolecular organization. Testing these predictions by combining surface-density measurements, mobility assays, residue-specific structural probes, and seeding experiments would directly establish when membrane enrichment, kinetic restriction, and amyloid-associated contact organization remain coupled and when they become separated.

By separating membrane interfacial recruitment from predefined membrane-bound-like helical ordering, this study reveals a staged regulation of αSyn assembly by an anionic POPC/POPG bilayer. In solution, both IDP_50_ and Hel_50_ formed condensates, but predefined helical/ordered segments increased local condensate density and favored intermediate-cluster capture while weakening global condensate cohesion. At the membrane, N-terminal anchoring enriched αSyn at the bilayer interface and supported flattened surface-associated condensates. However, membrane association also increased the population of proteins outside the largest condensate, indicating redistribution among the largest condensate, smaller membrane-associated populations, and the surrounding phase rather than simple stabilization of a macroscopic dense-phase.

Membrane association further slowed collective condensate dynamics and enhanced subdiffusive motion. Predefined helical/ordered segments reduced the displacement magnitude in solution but did not independently enhance subdiffusivity. Importantly, this kinetic restriction did not produce longer-lived registry-resolved NAC contacts, including parallel-like or antiparallel-like arrangements, or systematically stabilize persistent amyloid-like intermolecular organization. Together, these results show that membrane-driven surface assembly and condensate-scale kinetic restriction can be decoupled from persistent amyloid-like intermolecular organization. This decoupling provides a mechanistic basis for how lipid membranes can promote prominent αSyn surface assemblies without necessarily driving amyloid-competent maturation.

## 4 Materials and Methods

### 4.1 Coarse-Grained MD Modeling

Coarse-grained models of α-synuclein were constructed using the micelle-bound NMR structure of human αSyn (PDB ID 1XQ8) as the ordered state reference.^25^ Two molecular representations were considered. In the fully disordered representation, denoted IDP, the entire 140-residue sequence was modeled as an intrinsically disordered protein. In the partially helical representation, denoted Hel, residues 1–95 were retained in a helical/ordered state, whereas residues 96–140 were treated as disordered. For the Hel model, the native interruption within the 1–95 helical region present in the initial structure was explicitly preserved to maintain local flexibility at this site. Accordingly, elastic-network connections were not introduced across this break, and the elastic-network was applied only within the continuous structured segments. Topologies were generated using martinize2/vermouth with the Martini 3-IDP force field and mkdssp-based secondary-structure assignment.^61–65^ For the structured region, an elastic-network of standard recommended settings was applied with a force constant of 700 kJ mol^–1^ nm^–2^ and a cutoff distance of 0.85 nm.^66^ The -scFix option was used to restrain side-chain orientations in the structured region based on the reference all-atom structure.^67,68^ Lipid bilayers were generated using the INSANE script,^69^ with lateral dimensions of 48.42 × 48.42 nm^2^ and a lipid composition of 1-palmitoyl-2-oleoyl-*sn*-glycero-3-phosphocholine (POPC) and 1-palmitoyl-2-oleoyl-*sn*-glycero-3-phospho-(1′-rac-glycerol) (POPG) at a 7:3 ratio.

### 4.2 System Construction

Eight systems were constructed and named according to conformational state, chain number, and membrane association. The soluble monomer systems, IDP_1_ and Hel_1_, contained one fully disordered chain or one partially helical chain, respectively, in a cubic box of 20.0 × 20.0 × 20.0 nm^3^. The soluble 50-chain systems, IDP_50_ and Hel_50_, contained 50 fully disordered chains or 50 partially helical chains in cubic boxes of 48.42 × 48.42 × 48.42 nm^3^, corresponding to an αSyn concentration of 750 μM.

The membrane-associated 50-chain systems were generated by placing a lipid bilayer beneath initially dispersed protein chains along the *z*-direction while keeping the overall protein concentration approximately consistent with that in the corresponding soluble systems. The effective concentration of the membrane systems was calculated using the protein-accessible aqueous volume above the bilayer rather than the total simulation box volume. The resulting simulation box was approximately 48.42 × 48.42 × 54.00 nm^3^. The corresponding membrane-associated monomer systems, IDP_1_-Mem and Hel_1_-Mem, used the single-chain representations with the same bilayer composition. The bilayer consisted of POPC and POPG at a 7:3 molar ratio to represent an anionic membrane environment. All systems were solvated and ionized with NaCl to a final salt concentration of 0.15 M.

Two membrane-associated 50-chain systems were considered. The first, Hel_10_IDP_40_-Mem, contained 40 fully disordered chains and 10 partially helical chains. In this system, the 10 Hel chains were initially positioned at the membrane surface to mimic a heterogeneous membrane-associated environment in which surface-bound structured species coexist with disordered proteins. To maintain membrane proximity without rigid immobilization, these Hel chains were subjected to an additional layer-type flat-bottom positional restraint along the *z*-direction, referenced to *z* = 0, with a width parameter of *r*_fb_ = 6 nm. This restraint limited excessive displacement away from the membrane region while preserving lateral diffusion and local conformational flexibility. The second, IDP_50_-Mem, contained 50 fully disordered chains and served as a reference for comparison with both the solution-phase assemblies and the mixed membrane-associated system. Two membrane-associated monomer systems, IDP_1_-Mem and Hel_1_-Mem, were also simulated to provide a baseline for understanding how membrane association affects the conformational ensemble and dynamics of individual chains.

In addition, an inverted flat-bottom potential referenced to *z* = 0 was introduced to prevent protein chains in the aqueous phase from crossing the upper box boundary through periodic wrapping and binding to the periodic image of the lower membrane leaflet.^70^ This setup reduced artifacts arising from the mirrored lower leaflet at the top of the simulation box and helped preserve a more consistent bulk-like solvent environment and effective protein concentration above the membrane. The potential was defined by *r*_fb_ = −0. Three nm and *k*_fb_ = 10,000 kJ mol^–1^ nm^–2^, where the negative sign denotes the inverted form. It acted only on protein backbone beads and lipid headgroup beads, whereas water beads and ions remained unrestrained. Thus, although both the membrane-localizing restraint and the inverted flat-bottom potential were referenced to *z* = 0, they served distinct purposes: the former maintained membrane association of selected chains, whereas the latter suppressed periodic wraparound and spurious interactions with the mirrored lower leaflet.

### 4.3 Molecular Dynamics Simulations

All simulations were performed using GROMACS 2022.5 under periodic boundary conditions.^71^ Nonbonded interactions were evaluated using the Verlet cutoff scheme, with reaction-field electrostatics using a relative dielectric constant of 15 and a cutoff distance of 1.1 nm, and van der Waals interactions using the same cutoff distance. Temperature was maintained at 310 K using the velocity-rescaling thermostat.^72^ Isotropic pressure coupling was applied to soluble systems, whereas semi-isotropic pressure coupling was used for membrane-associated systems; the Parrinello–Rahman barostat was used for pressure control.^73^

Production simulations were carried out for 10 μs for the monomer systems (IDP_1_, Hel_1_, IDP_1_-Mem, and Hel_1_-Mem). For the multichain systems, IDP_50_, Hel_50_, and Hel_10_IDP_40_-Mem were simulated for 20 μs. In these systems, most protein chains assembled into a large condensate within approximately the first 10 μs. To focus the quantitative analyses on the resulting late-stage condensed states rather than on the initial assembly process, only the final 10 μs of these trajectories were used for the analyses reported in this work.

The IDP_50_-Mem system was used primarily as a simplified reference for assessing the effect of membrane association on a fully disordered multichain assembly. In preliminary simulations initiated from spatially dispersed chains, the proteins spontaneously accumulated at the membrane surface and formed a large membrane-associated cluster after 10 μs. Having established this spontaneous assembly behavior, three independent production trajectories were initiated from a preformed membrane-associated condensate and simulated for 10 μs. The full 10 μs trajectories were used for analysis. Each system was simulated in three independent replicates with different initial states.

## 5 Analysis

Trajectory analyses were carried out using in-house Python scripts based on MDAnalysis.^74,75^ Because many observables were evaluated after identifying the largest condensate, we first define the common cluster selection convention and then give compact definitions of each observable.

### 5.1 Cluster Identification and Sampling Convention

For multichain systems, condensates were identified from a chain-level contact graph. Unless otherwise specified, a cutoff distance of *d*_*c*_ = 8 Å was used to define interchain contacts. At each frame *t*, each protein chain was treated as one node in the graph. Two different chains *m* and *n* were connected by an edge when the minimum distance between their backbone beads was less than *d*_*c*_. The minimum interchain backbone distance was defined as

$$d_{\mathrm{mn}}^{\min}\left(t\right)=\min_{a\in m,b\in n}\left|r_{a}^{\mathrm{BB}}\left(t\right)-r_{b}^{\mathrm{BB}}\left(t\right)\right|$$

where *a* and *b* denote backbone beads from chains *m* and *n*, respectively. The chain-contact matrix was then defined as

$$A_{\mathrm{mn}}\left(t\right)=\{\begin{array}{ll} 1, & m\neq n\;\mathrm{and}\;d_{\mathrm{mn}}^{\min}\left(t\right)<d_{c},\\ 0, & \mathrm{otherwise}. \end{array}$$

Thus, *A*_*mn*_(*t*) = 1 indicates that chains *m* and *n* are directly connected in the contact graph at frame *t*, whereas *A*_*mn*_(*t*) = 0 indicates that they are not directly connected. Self-connections were not considered, so *A*_*mm*_(*t*) = 0.

Instantaneous clusters were defined as the connected components of this chain-contact graph. In this definition, chains belong to the same cluster if they are connected either directly or through a continuous path of intermediate chain–chain contacts. For example, if one chain contacts a second chain, and the second chain contacts a third chain, then all three chains are assigned to the same cluster even if the first and third chains are not in direct contact. Among all instantaneous clusters at frame *t*, the one containing the largest number of chains was defined as the frame-wise largest cluster, *C*_L_(*t*). Its size was

$$N_{\max}\left(t\right)=\left|C_{L}\left(t\right)\right|$$

Static condensate properties were calculated from the frame-wise largest cluster, *C*_L_(*t*).

For dynamical analyses, we used only the chains that remained in the largest cluster throughout the analysis window T . This persistent chain set was defined as

$$S_{T}=\underset{t\in T}{\cap }C_{L}\left(t\right)$$

This definition prevents MSD and other dynamical observables from being dominated by chain exchange between the condensate and the surrounding dilute-phase.

The same 8 Å cutoff was used to identify the clusters used in cluster growth, shape-descriptor, neighbor-counting, MSD, and contact-map analyses unless otherwise specified. When an observable was reported for a specific chain subset, such as the fully disordered chains in the mixed membrane system, the subset filter was applied after cluster selection. Reported observables and distributions were pooled over independent replicates.

### 5.2 Mean-Squared Displacement

Before the MSD calculations, trajectories were made whole across periodic boundaries and each frame was translated so that the center of mass of the frame-wise largest condensate was located at the origin. The resulting MSD therefore excludes translational motion of the condensate and reports chain motion relative to the condensate center.

For chain-level dynamics, the position of chain *m* was represented by its bead-averaged center

$$R_{m}\left(t\right)=\frac{1}{N_{m}}\sum_{a\in m}r_{a}\left(t\right)$$

where *N*_*m*_ is the number of beads in chain *m*. For condensate-internal dynamics, MSDs were calculated over the persistent chain set $S_{T}$ , defined as the chains that remained in the largest cluster throughout the analysis window

$$\mathrm{MSD}\left(\tau \right)={\left\langle {\left|R_{m}\left(t+\tau \right)-R_{m}\left(t\right)\right|}^{2}\right\rangle }_{m,t}$$

The membrane-plane mean-squared displacement was calculated from the projection of the chain-center displacement onto the bilayer plane

$${\mathrm{MSD}}_{\mathrm{xy}}\left(\tau \right)={\left\langle {\left[R_{m,x}\left(t+\tau \right)-R_{m,x}\left(t\right)\right]}^{2}+{\left[R_{m,y}\left(t+\tau \right)-R_{m,y}\left(t\right)\right]}^{2}\right\rangle }_{m,t}$$

The average was taken over the selected chains and over all time origins for which both *t* and *t* + τ were within the analysis window. For monomer systems, the same expression was applied to the single-chain without cluster filtering. When state-resolved MSDs were required, the average was restricted to continuous residence intervals of at least 1 μs. The anomalous diffusion exponent was obtained by fitting

$$\mathrm{MSD}\left(\tau \right)=K\tau^{\alpha }$$

over lag times from 10 to 100 ns on a log–log scale.

### 5.3 Shape Anisotropy and Principal Axes

For each frame, $A_{L}\left(t\right)$ denotes all beads belonging to chains in *C*_L_(*t*). The gyration tensor of that cluster was calculated as

$$T\left(t\right)=\frac{1}{\sum_{a\in A_{L}\left(t\right)}M_{a}}\sum_{a\in A_{L}\left(t\right)}M_{a}\left(r_{a}-r_{\mathrm{com}}\right){\left(r_{a}-r_{\mathrm{com}}\right)}^{T}$$

where *M*_*a*_ is the mass of bead *a*, and **r**_com_ is the center of mass of $A_{L}\left(t\right)$ . Its eigenvalues were denoted by λ_1_ ≤ λ_2_ ≤ λ_3_. The cluster radius of gyration and principal axis lengths were then defined as

$$R_{g}^{2}=\lambda_{1}+\lambda_{2}+\lambda_{3},L_{\mathrm{minor}}=\sqrt{\lambda_{1}},L_{\mathrm{mid}}=\sqrt{\lambda_{2}},L_{\mathrm{major}}=\sqrt{\lambda_{3}}$$

The relative shape anisotropy parameter was calculated as

$$\kappa^{2}=1-\frac{3\left(\lambda_{1}\lambda_{2}+\lambda_{2}\lambda_{3}+\lambda_{3}\lambda_{1}\right)}{{\left(\lambda_{1}+\lambda_{2}+\lambda_{3}\right)}^{2}}$$

### 5.4 Radial Mass Density Profiles and Interfacial Fit

Radial mass density profiles were calculated after translating each frame so that the center of mass of the frame-wise largest condensate was placed at the origin. The radial coordinate was divided into concentric spherical shells indexed by *k*, with the *k*th shell spanning the radial interval from *r*_*k*_ to *r*_*k*+1_. Protein beads were assigned to the corresponding shells at each analyzed frame, and the volume of the *k*th shell was calculated as

$$V_{k}=\frac{4\pi }{3}\left(r_{k+1}^{3}-r_{k}^{3}\right)$$

Mass densities were calculated by summing the bead masses in each shell, dividing by the shell volume, and averaging over the analyzed frames. Only shells within the maximum valid radial range allowed by the instantaneous periodic-box geometry were included in the fitting and quantitative analysis. The maximum valid radius was defined as one-half of the shortest instantaneous box dimension, thereby avoiding periodic boundary artifacts. In Figure S11, radial distances beyond the corresponding valid boundary are indicated by gray shading.

The overall radial mass density of the condensate, ρ­(*r*), was calculated using all protein beads and fitted with a hyperbolic-tangent interfacial model, following previous analyses of biomolecular condensate interfaces.^76,77^ The fitted function was

$$\rho \left(r\right)=\frac{\rho_{\mathrm{dense}}+\rho_{\mathrm{dilute}}}{2}-\frac{\rho_{\mathrm{dense}}-\rho_{\mathrm{dilute}}}{2}\tanh\left(\frac{2\left(r-R_{c}\right)}{w}\right)$$

where ρ_dense_ and ρ_dilute_ are the fitted dense- and dilute-phase plateau densities, respectively, *R*_*c*_ is the effective condensate radius defined by the midpoint of the fitted density transition, and *w* is the interfacial width.

The density-derived apparent transfer free energy was calculated directly from the two fitted plateau densities

$$\Delta G_{\mathrm{app}}=-\mathrm{RT}\ln\left(\frac{\rho_{\mathrm{dense}}}{\rho_{\mathrm{dilute}}}\right)$$

where *R* is the gas constant and *T* is the simulation temperature.^78,79^

For the quantitative comparison in Figure 3D, the radial density profile of each replicate trajectory was fitted independently, and the resulting Δ*G*_app_ values were reported as mean ± SD across the three replicates. For visualization of the overall interfacial profile, Figure S11 shows a fit to the replicate-averaged density profile. The values annotated in Figure S11 therefore summarize the fitted replicate-averaged profiles, whereas Figure 3D reports the statistics of the independently fitted replicate trajectories.

Segment-resolved radial mass density profiles were calculated using the same origin, radial shells, and normalization. For each sequence segment, all beads belonging to that segment from all chains were pooled, and their mass density was computed in each shell. These segment-resolved profiles were averaged over analyzed frames and independent replicates to quantify how different sequence regions contribute to the radial mass distribution of the condensate.

### 5.5 Contact Maps

Residue–residue contact maps were calculated using a smooth switching function

$$s\left(r\right)=\frac{1}{2}\left[1-\tanh\left(\beta \left(r-d_{0}\right)\right)\right]$$

where *d*_0_ = 8 Å and β = 1 Å^–1^. This function gives values close to 1 for contacting residue pairs and values close to 0 for distant residue pairs.

For intrachain contact maps, the distance *r*_*ij*_ was measured between the backbone beads of residues *i* and *j* within the same chain. The instantaneous intrachain contact score was defined as

$$c_{\mathrm{ij}}^{\mathrm{intra}}=s\left(r_{\mathrm{ij}}\right)$$

The reported intrachain contact maps were obtained by averaging *c*_*ij*_^intra^ over all analyzed frames and over the selected chains. For single-chain systems, the average was taken over the single-chain. For multichain systems, only chains belonging to the frame-wise largest cluster *C*_L_(*t*) were included.

For interchain contact maps, residue *i* from one chain and residue *j* from another chain were considered. For a chain pair *m* and *n*, the instantaneous interchain contact score was calculated as

$$c_{\mathrm{ij}}^{\mathrm{inter}}=\frac{1}{2}\left[s\left(r_{\mathrm{ij}}^{\mathrm{mn}}\right)+s\left(r_{\mathrm{ji}}^{\mathrm{mn}}\right)\right]$$

where *r*_*ij*_^*mn*^ is the distance between residue *i* in chain *m* and residue *j* in chain *n*, and *r*_*ij*_^*mn*^ is the distance between residue *j* in chain *m* and residue *i* in chain *n*. This symmetrization removes the arbitrary ordering of two identical chains.

The reported interchain contact maps were averaged over all analyzed frames and over directly contacting chain pairs within the frame-wise largest cluster. A chain pair was included when both chains belonged to *C*_L_(*t*) and their minimum interchain backbone bead distance was less than 8 Å.

### 5.6 NAC Axial-Packing and Anti-Diagonal Registry Analysis

Directional NAC packing was analyzed for soluble IDP_50_ and Hel_50_ simulations using the full 0–20 μs trajectories sampled every 10 ns from three independent trajectories per condition. Each chain contained 140 backbone beads, and the NAC region was defined as residues 61–95. At each sampled frame, clusters were identified from a chain-contact graph in which two chains were connected if any interchain backbone bead pair had a separation less than 8 Å. Directional-packing metrics were then calculated only for chain pairs with a direct NAC–NAC contact, defined as at least one interchain NAC backbone bead pair with a separation less than 8 Å.

For each directly NAC-contacting chain pair, NAC coordinates were unwrapped across periodic boundaries, and the principal axis of each NAC segment was obtained by PCA. Because PCA axes are sign-degenerate, each axis was oriented from N to C using the vector connecting the mean positions of the first and last four NAC beads. The signed relative orientation was calculated as

$$\cos\theta_{\mathrm{mn}}=u_{m}^{\mathrm{NAC}}\cdot u_{n}^{\mathrm{NAC}}$$

where **u**_*m*_^NAC^ and **u**_*n*_^NAC^ are the N-to-C-oriented NAC axes of chains *m* and *n*. The signed cos θ distribution was obtained by binning values from −1 to 1 into 40 bins. Counts were normalized within each trajectory, and the mean probability per bin and SD were calculated across three independent trajectories. A NAC-contacting pair was classified as axially aligned when |cos θ_*mn*_| ≥ 0.5, and the aligned-pair percentage was calculated as

$$f_{\mathrm{aligned}}=100\times \frac{N\left(\left|\cos\theta \right|\geq 0.5\right)}{N_{\mathrm{NAC}‐\mathrm{contacting}\;\mathrm{pairs}}}$$

To quantify broad head-to-tail-like NAC registry, all pairwise NAC backbone distances *d*_*ij*_ within each directly NAC-contacting chain pair were converted to the same smooth contact score *s*(*d*_*ij*_) defined above for the contact-map analysis. Here, *i* and *j* denote the residue indices of the two NAC beads. The parallel-register band was centered on the contact-map diagonal, *i* = *j*. The antiparallel or head-to-tail band was centered on the antidiagonal, *i* + *j* = 156, where 156 = 61 + 95 corresponds to the reverse sequence pairing of the first and last NAC residues. To allow limited registry shifts, registry displacements of fewer than eight residue positions were permitted. Thus, the antidiagonal and parallel-register bands were defined as

|i+j−156|<8and|i−j|<8

respectively. Antidiagonal preference was calculated as

$$P_{\mathrm{anti}}=100\times \left(\frac{\left\langle s_{\mathrm{anti}}\right\rangle }{\left\langle s_{\mathrm{parallel}}\right\rangle }-1\right)$$

where ⟨*s*_anti_⟩ and ⟨*s*_parallel_⟩ are the mean smooth contact scores in the antidiagonal and parallel-register bands. Positive values indicate stronger antidiagonal than parallel-register contacts. All metrics were calculated within each independent trajectory first, and means and SDs were then calculated across the three trajectory-level values.

### 5.7 Membrane-Normal Segment Distribution

The membrane midplane was defined from the center-of-mass *z* coordinate of the PO4 headgroups. For each protein chain, the center-of-mass position of each sequence segment was calculated, and its displacement along the membrane-normal direction was measured relative to the membrane midplane. Positive and negative values therefore indicate positions on opposite sides of the membrane midplane. The resulting membrane-normal displacements were collected over all chains, frames, and independent replicates, and the corresponding distributions were estimated using kernel-density estimation.

### 5.8 Inter-Chain Neighbor Distribution

Local coordination within the condensate was quantified using the same chain-level contact graph defined above with an 8 Å cutoff. For each chain in the frame-wise largest cluster, we counted the number of other chains in the same cluster that were directly connected to it in the contact graph. This number was defined as the local number of neighboring chains for that frame. Neighbor counts from all chains, frames, and independent replicates were pooled to obtain the probability distribution of local coordination numbers.

### 5.9 Surface Tension

Surface tension was estimated from capillary-like shape fluctuations of the largest particle cluster, following the bead-level mass-distribution treatment of Benayad et al.^79^ Protein coordinates were first made whole across periodic boundaries. At each frame, particles were connected if their separation was less than 8 Å, and the largest connected particle cluster was selected. Because covalently linked beads within each protein remain connected at this cutoff, this procedure retains molecular connectivity while defining the condensate boundary from its bead-level spatial distribution. The covariance tensor of its coordinates gave eigenvalues λ_1_ ≤ λ_2_ ≤ λ_3_. The instantaneous equivalent radius was

$$R\left(t\right)=\sqrt{5}{\left[\lambda_{1}\left(t\right)\lambda_{2}\left(t\right)\lambda_{3}\left(t\right)\right]}^{1/6}$$

After block averaging, the mean radius *R* was used to define semiaxes that preserve the instantaneous shape anisotropy

$$a=R\frac{\lambda_{1}^{1/3}}{{\left(\lambda_{2}\lambda_{3}\right)}^{1/6}},b=R\frac{\lambda_{2}^{1/3}}{{\left(\lambda_{1}\lambda_{3}\right)}^{1/6}},c=R\frac{\lambda_{3}^{1/3}}{{\left(\lambda_{1}\lambda_{2}\right)}^{1/6}}$$

With δ*a* = *a* – *R*, δ*b* = *b* – *R*, and δ*c* = *c* – *R*, the two fluctuation amplitudes were

$${\mathrm{var}}_{+}=\frac{\langle {\left(\delta a+\delta b\right)}^{2}\rangle +\left\langle {\left(\delta b+\delta c\right)}^{2}\right\rangle +\left\langle {\left(\delta c+\delta a\right)}^{2}\right\rangle }{3}$$

$${\mathrm{var}}_{-}=\frac{\langle {\left(\delta a-\delta b\right)}^{2}\rangle +\left\langle {\left(\delta b-\delta c\right)}^{2}\right\rangle +\left\langle {\left(\delta c-\delta a\right)}^{2}\right\rangle }{3}$$

After converting var. _±_ from Å^2^ to m^2^, the quadrupolar tension estimates were

$$\gamma_{20}=\frac{5k_{B}T}{16\pi {\mathrm{var}}_{+}},\gamma_{22}=\frac{15k_{B}T}{16\pi {\mathrm{var}}_{-}}$$

The reported surface tension was the mean of these two estimates

$$\gamma =\frac{\gamma_{20}+\gamma_{22}}{2}$$

### 5.10 Continuous NAC Contact Survival Analysis

To quantify the uninterrupted residence times of interchain NAC contacts, we identified individual continuous contact episodes and calculated their empirical survival functions. The NAC region was defined as residues 61–95, and contacts were evaluated between backbone beads using a distance cutoff of *r*_*c*_ = 0.8 nm. The analysis was performed over the same final-10 μs window and trajectory sampling interval used for the condensate dynamics analysis.

NAC contacts were evaluated only for chain pairs drawn from the persistent largest-cluster chain set $S_{T}$ , defined above as the chains that remained in the frame-wise largest cluster throughout the analysis window. No additional distance cutoff from the condensate center was applied. When a chain subset was specified, such as the fully disordered chains in Hel_10_IDP_40_-Mem, only chains belonging to both that subset and $S_{T}$ were included.

For residues *i* and *j* in the NAC regions of chains *m* and *n*, respectively, the residue-pair contact indicator was defined as

$$x_{\mathrm{mn}}^{\mathrm{ij}}\left(t_{\xi }\right)=1\left[d_{\mathrm{mn}}^{\mathrm{ij}}\left(t_{\xi }\right)<r_{c}\right]$$

where *d*_*mn*_^*ij*^(*t*_ξ_) is the corresponding backbone–backbone distance at time *t*_ξ_, and **1**[·] denotes the indicator function.

For the most permissive definition, denoted Any BB–BB, a chain pair was considered to be in contact when any pair of NAC backbone beads satisfied the distance criterion

$$h_{\mathrm{mn}}^{\mathrm{any}}\left(t_{\xi }\right)=1\left[\min_{\begin{array}{l} i\in \mathrm{NAC}\\ j\in \mathrm{NAC} \end{array}}d_{\mathrm{mn}}^{\mathrm{ij}}\left(t_{\xi }\right)<r_{c}\right]$$

Registry-resolved contacts were classified into four groups. Strict parallel-like contacts required residues with the same sequence index, *j* = *i*, whereas strict antiparallel-like contacts required the reverse sequence correspondence *i* + *j* = 156, where 156 = 61 + 95. To allow limited registry shifts, banded parallel-like contacts were defined by |*i* – *j*| < 8, and banded antiparallel-like contacts by |*i* + *j* – 156| < 8. The banded definitions therefore correspond to finite-width regions around the diagonal and antidiagonal of the NAC contact-map, respectively.

For each registry class, two permissive contact modes were considered. The ≥1 criterion required at least one NAC residue on one chain to contact an allowed partner residue on the other chain, whereas the ≥3 criterion required at least three distinct NAC residues to participate in qualifying contacts. The three residues in the ≥3 definition were not required to be consecutive.

More restrictive contact modes required at least three consecutive NAC residues on each chain to form a consistently ordered segment. These criteria can be written compactly as

$$\prod_{k=0}^{2}x_{\mathrm{mn}}^{i+k,j+\sigma k}\left(t_{\xi }\right)=1$$

for at least one pair of valid NAC triplet starting positions *i* and *j*. Here, σ = +1 was used for strict parallel-like and banded parallel-like triplets, whereas σ = −1 was used for strict antiparallel-like and banded antiparallel-like triplets. For strict parallel-like triplets, *j* = *i*; for strict antiparallel-like triplets, *i* + *j* = 156. For the two banded definitions, the triplet starting positions instead satisfied |*i* – *j*|< 8 or |*i* + *j* – 156|< 8, respectively. Because the criterion is met by the presence of at least one qualifying triplet, longer consecutively ordered contact segments are also included. Thus, the strict criteria required exact forward or reverse sequence correspondence, whereas the banded criteria permitted a limited registry displacement while preserving the forward or reverse ordering of the contacting segment.

For each chain pair in $S_{T}$ and each contact mode, consecutive frames satisfying the corresponding binary contact criterion were grouped into one continuous contact episode. An episode ended as soon as the complete criterion was no longer satisfied, and a contact that broke and subsequently reformed was counted as a new event. Episodes that remained present at the end of the analysis window were recorded using their observed duration up to the final frame.

For each independent trajectory, the empirical survival function *S*(*t*) was calculated as the fraction of contact episodes with duration at least *t*. Survival curves were interpolated onto a common time grid and averaged across trajectories for visualization. Characteristic survival times were extracted directly from the empirical curves without exponential fitting. For each trajectory and contact mode, *T*_*S*_(1/*e*) and *T*_*S*_(0.1) were defined as the first times at which *S*(*t*) decreased to 1/*e* and 0.1, respectively. *T*_*S*_(1/*e*) characterizes the main decay of the contact-duration distribution, whereas *T*_*S*_(0.1) emphasizes its longer-lived tail. Reported values and error bars represent the mean and SD across the three trajectory-level values. A threshold was reported as not reached when the survival curve remained above it throughout the analyzed time range.

### 5.11 Kolmogorov–Smirnov Comparison of NAC Contact-Lifetime Distributions

To complement the characteristic survival-time metrics, the complete NAC contact-lifetime distributions were compared using the two-sample Kolmogorov–Smirnov (KS) statistic. The same continuous contact episodes and contact definitions described above for the NAC survival analysis were used.

For each system and contact definition, contact-lifetime episodes from the three independent trajectories were pooled to construct an empirical cumulative distribution function, *F*(*t*). Pairwise comparisons between systems *A* and *B* were quantified as

$$D_{\mathrm{KS}}=\max_{t}\left|F_{A}\left(t\right)-F_{B}\left(t\right)\right|$$

where *F*_*A*_(*t*) and *F*_*B*_(*t*) are the empirical cumulative distribution functions of the corresponding contact-lifetime distributions. Larger *D*_KS_ values indicate greater separation between the complete lifetime distributions.

Pairwise *D*_KS_ values were calculated among IDP_50_, Hel_50_, IDP_50_-Mem, and the IDP–IDP pairs in Hel_10_IDP_40_-Mem. This analysis provides a full-distribution comparison complementary to *T*_S_(1/*e*) and *T*_S_(0.1).

### 5.12 NAC Contact Autocorrelation Analysis

NAC contact autocorrelation functions were calculated from the same binary contact trajectories, *h*_*mn*_^(ν)^(*t*_ξ_), for chain pairs in $S_{T}$ . For contact mode ν in trajectory l , the normalized autocorrelation function was

$$C_{\nu }^{\left(l\right)}\left(t\right)=\frac{{\left\langle \left[h_{\mathrm{mn}}^{\left(\nu \right)}\left(t_{\xi }\right)-{\mathrm{h̅}}_{\mathrm{mn}}^{\left(\nu \right)}\right]\left[h_{\mathrm{mn}}^{\left(\nu \right)}\left(t_{\xi }+t\right)-{\mathrm{h̅}}_{\mathrm{mn}}^{\left(\nu \right)}\right]\right\rangle }_{m,n\in S_{T},\xi }}{{\left\langle {\left[h_{\mathrm{mn}}^{\left(\nu \right)}\left(t_{\xi }\right)-{\mathrm{h̅}}_{\mathrm{mn}}^{\left(\nu \right)}\right]}^{2}\right\rangle }_{m,n\in S_{T},\xi }}$$

where *h̅*_*mn*_^(ν)^ is the time-averaged contact-state for chains *m* and *n*, and the average was taken over included chain pairs and all valid time origins. Chain pairs with no change in contact-state during the analysis window were excluded from the autocorrelation average. A faster decay indicates more rapid rearrangement of the corresponding NAC contact mode.

For each independent trajectory and contact mode, *T*_ACF_(1/*e*) and *T*_ACF_(0.1) were defined as the first times at which the normalized autocorrelation function decreased to 1/*e* and 0.1, respectively. Reported *T*_ACF_(0.1) and *T*_ACF_(1/*e*) values and error bars are the mean and SD across the three trajectory-level values. A threshold was reported as not reached when the autocorrelation function remained above the corresponding threshold throughout the analyzed time range.

## Supplementary Material



## Data Availability

Processed data presented in the figures, together with simulation input files and analysis scripts, are publicly available in the GitHub repository at https://github.com/JChen901/aS-Condensate-on-Membrane.

## Notes

Analyses for soluble and membrane-associated αSyn systems, including largest-cluster growth, residue-level contact maps, NAC axial-packing and antidiagonal registry analyses, membrane-normal protein distributions, membrane-contact frequencies, initial membrane-system configurations, condensate shape analysis, radial density profiles, dense- and dilute-phase density comparisons, raw MSD curves used to estimate anomalous diffusion exponents, empirical survival functions under strict and banded parallel-like and antiparallel-like NAC contact definitions, characteristic continuous contact-survival times at *S* = 1/*e* and *S* = 0.1, NAC contact-state autocorrelation times, registry-window sensitivity, full-distribution Kolmogorov–Smirnov comparisons, and pair-type-resolved NAC contact persistence (PDF)
