# Supplementary material for: Membranes Decouple α‑Synuclein Surface Assembly and Kinetic Restriction from Persistent Amyloid-like Intermolecular Organization
Source: JACS Au. 2026 Sep 24;6(10):5917–34. doi: 10.1021/jacsau.6c01222 (PMC13714835; doi:10.1021/jacsau.6c01222)
Supplement: Supplementary file 1 [file au6c01222_si_001.pdf]

Supplementary Information

Membranes decouple α-synuclein surface assembly and kinetic restriction from persistent amyloid-like intermolecular organization

Jinyu Chen[1] and Xiakun Chu[1,2,3,*]

[1]Advanced Materials Thrust, Function Hub,
The Hong Kong University of Science and Technology (Guangzhou),
Guangzhou, Guangdong, 511453, China

[2]Guangzhou Municipal Key Laboratory of Materials Informatics,
The Hong Kong University of Science and Technology (Guangzhou),
Guangzhou, Guangdong, 511453, China

[3]Division of Life Science,
The Hong Kong University of Science and Technology,
Clear Water Bay, Kowloon, Hong Kong SAR 999077, China

[*]Correspondence: xiakunchu@hkust-gz.edu.cn

Supplementary Figures

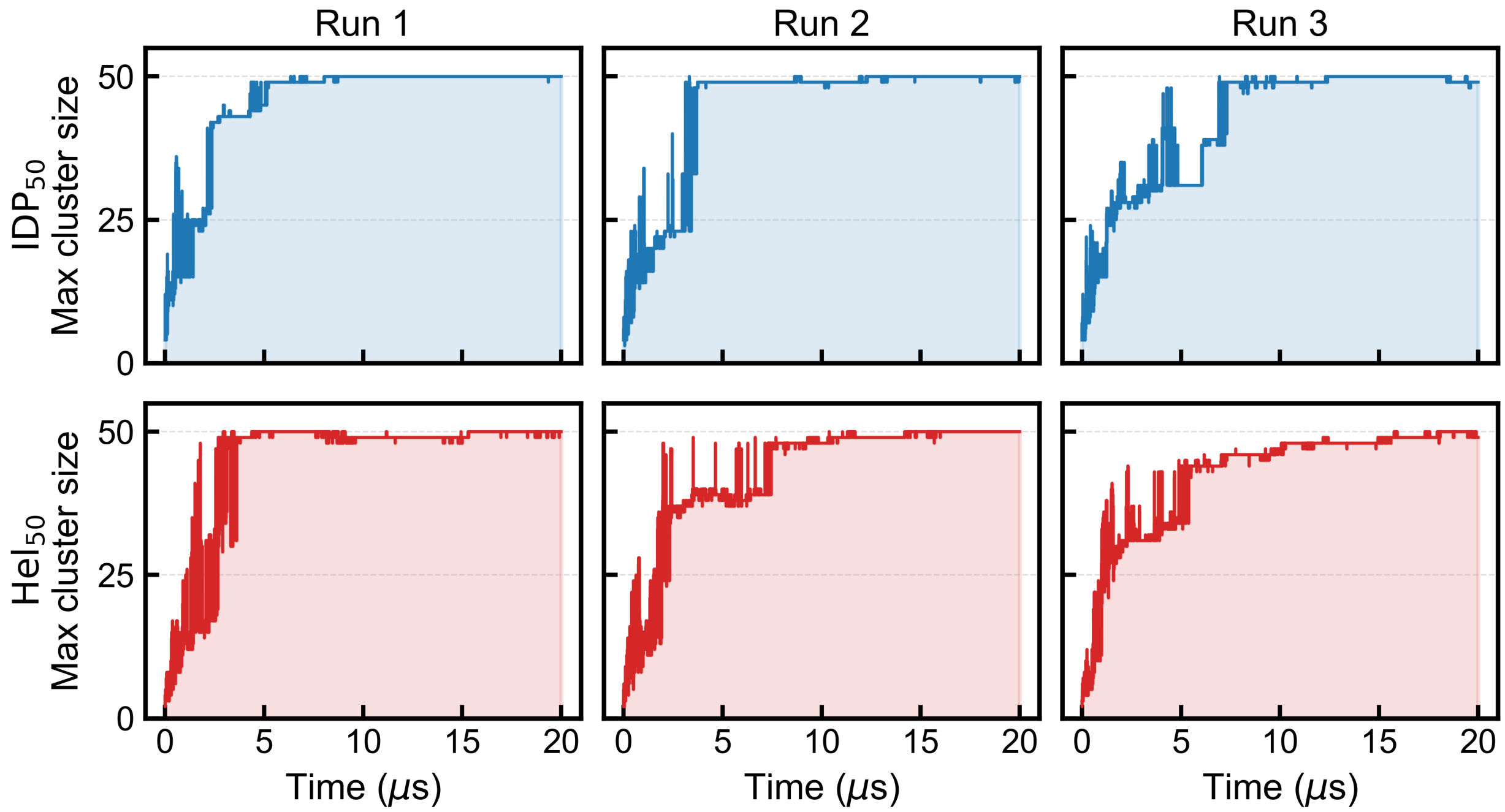

Figure S1: Largest-cluster growth in three independent soluble 50-chain simulations. Time traces show the size of the largest chain-level cluster, N_{max}, for IDP_{50} (top row, blue) and Hel_{50} (bottom row, red). The dashed horizontal lines mark the incorporation of half of the system, 25 chains, and the complete incorporation of all 50 chains into the largest condensate.

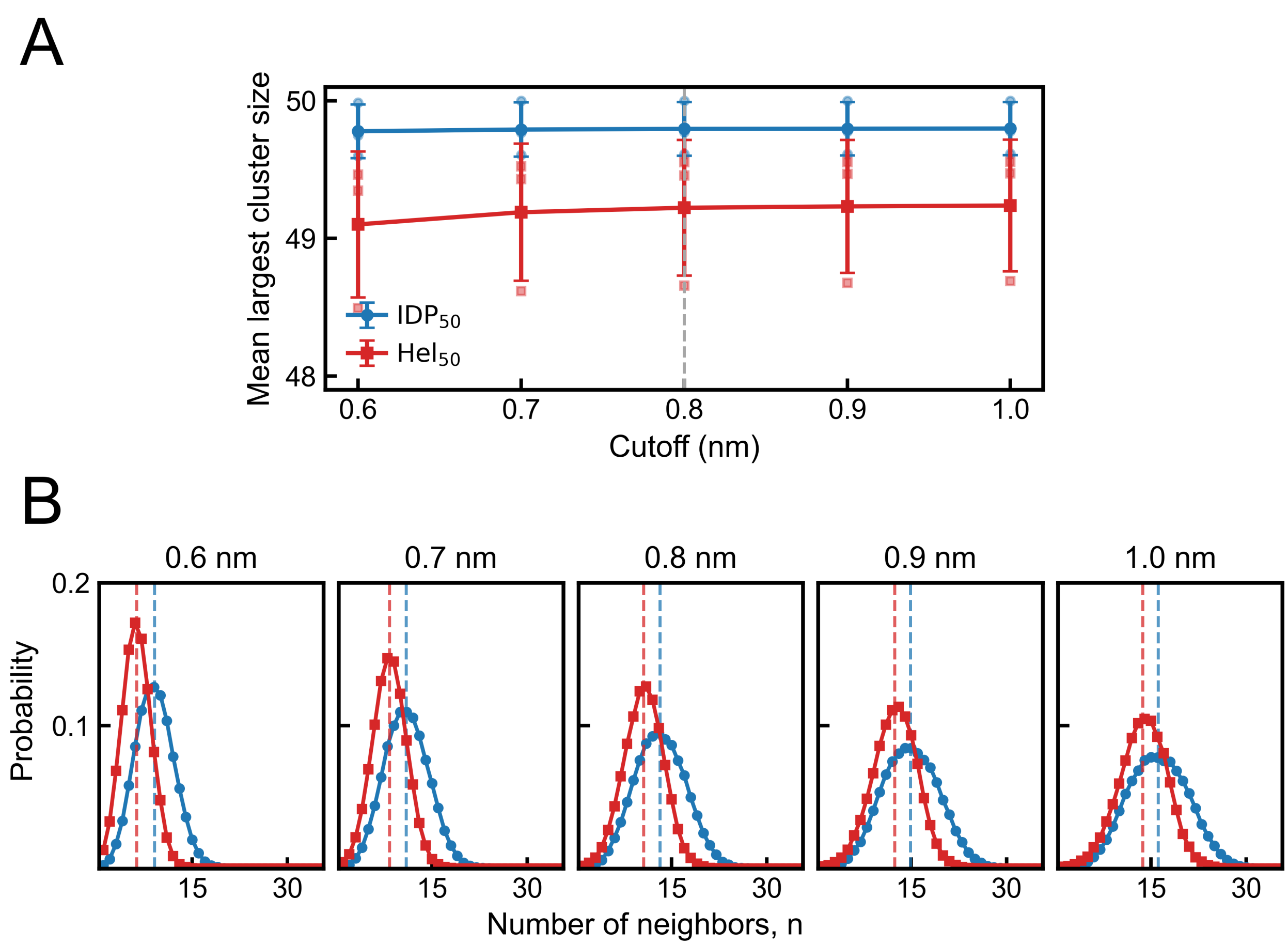

Figure S2: Sensitivity of intermolecular contact analysis to the distance cutoff. Two chains are considered in contact if any pair of their backbone beads is separated by less than the specified cutoff. (A) Mean size of the largest connected cluster at cutoffs of 0.6–1.0 nm. Solid symbols and error bars represent the mean and standard deviation across three independent simulations; faint symbols indicate individual simulation means. (B) Distributions of the number of neighboring chains within the largest cluster, pooled over sampled chains and frames from the three simulations. Dashed vertical lines indicate the corresponding mean neighbor numbers. Blue and red denote IDP_{50} and Hel_{50}, respectively. Results use the final 10 μs of each simulation.

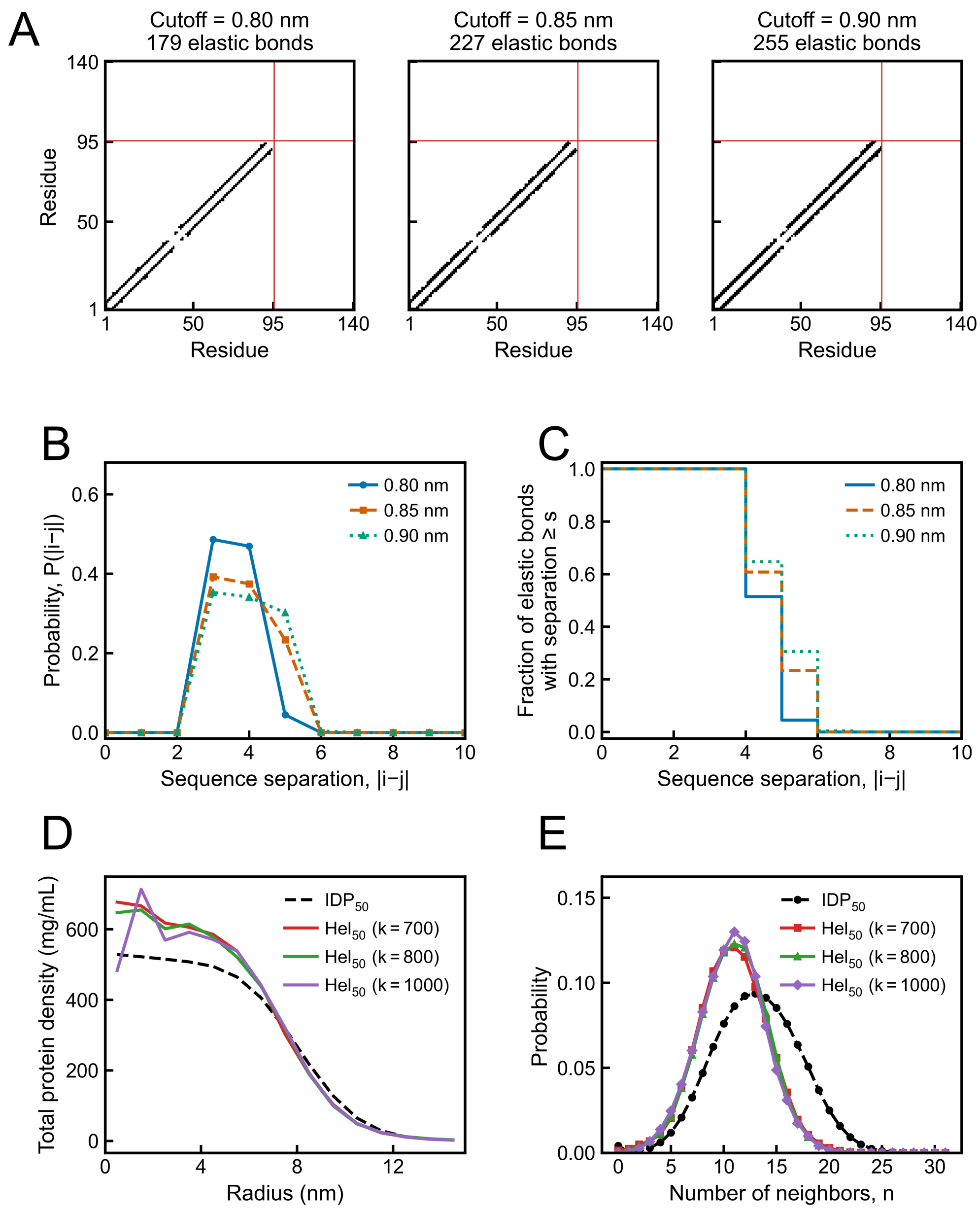

Figure S3: Elastic-network topology and sensitivity of condensate properties to elastic-network parameters. (A) Elastic-network bond maps constructed using cutoff distances of 0.80, 0.85, and 0.90 nm, respectively. Red lines mark the boundary of the elastic-network region at residue 95. (B) Probability distributions of the sequence separation, $|i - j|$, of elastic-network bonds for the three cutoff values. (C) Fraction of elastic-network bonds with sequence separation greater than or equal to s. (D) Radial total protein-density profiles of IDP50 and Hel50 simulated using elastic-network force constants of $K = 700$, 800, and 1000 kJ mol^{-1} nm^{-2}. (E) Corresponding distributions of the number of intermolecular neighboring chains, n, for IDP$_{50}$ and the three Hel$_{50}$ elastic-network strengths. Panels D and E were obtained from two independent 1.5 μs sensitivity trajectories for each elastic-network force constant, initiated from the final frame of the original Hel$_{50}$ trajectory. All other simulation conditions were kept consistent with those used in the main simulations.

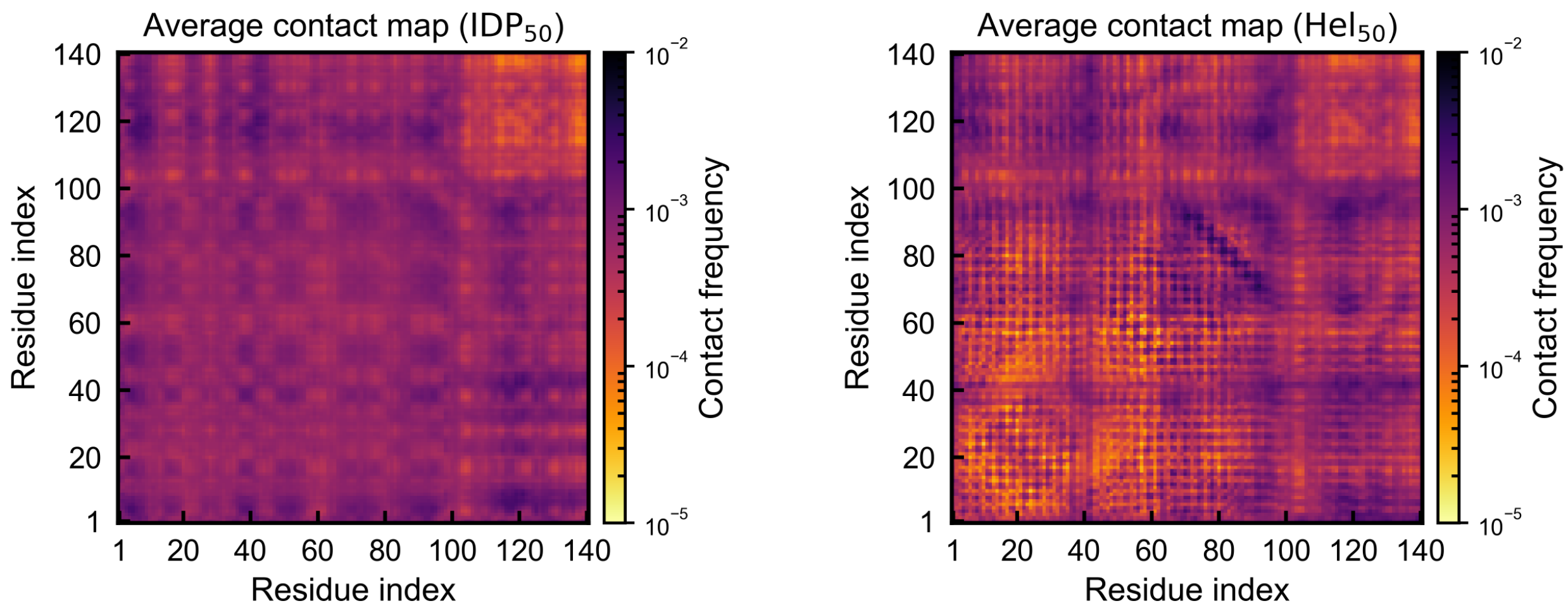

Figure S4: Average inter-chain contact-frequency maps of soluble IDP$_{50}$ and Hel$_{50}$ condensates. The residue-level maps were averaged over directly contacting chain pairs in the frame-wise largest cluster, analyzed frames, and three independent replicate trajectories. Both panels use the same logarithmic color scale. These absolute contact patterns were used to calculate the contact-frequency difference map shown in Fig. 1D.

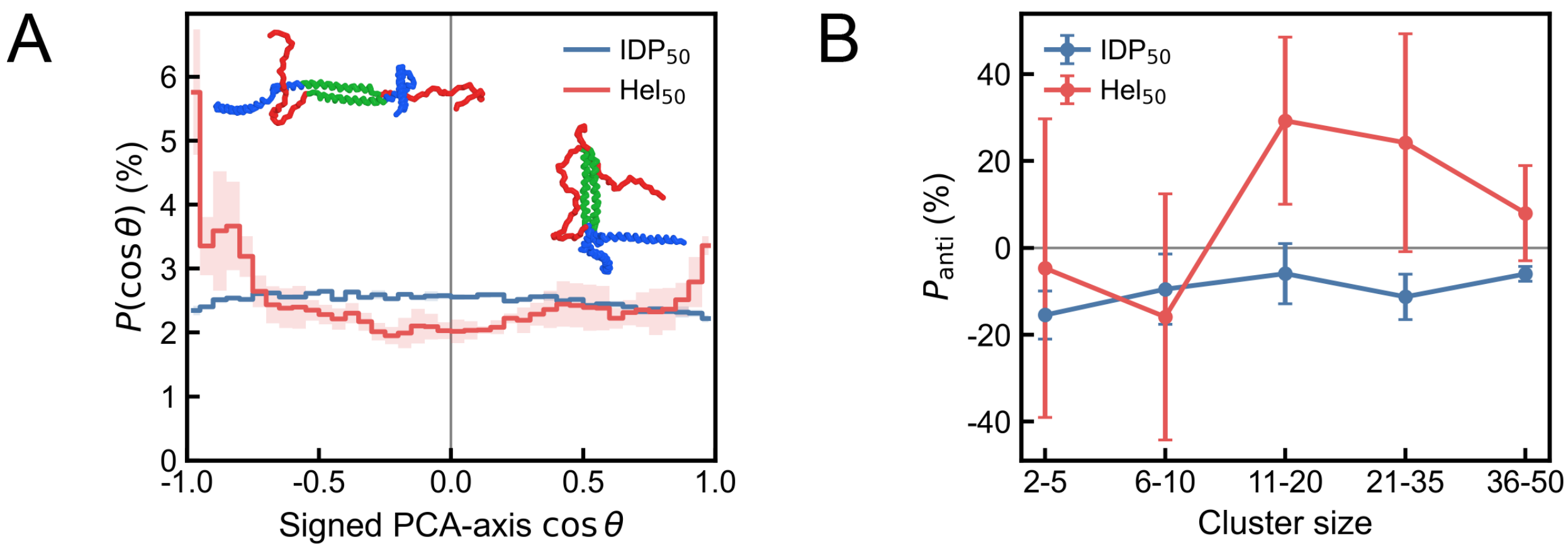

Figure S5: Auxiliary analysis of NAC axial orientation and anti-diagonal contact enrichment. (A) Signed PCA-axis $\cos\theta$ distribution for directly NAC-contacting chain pairs. The NAC principal axis was calculated from residues 61–95 and oriented from N to C. Values near $+1$ and -1 indicate parallel-like and antiparallel-like axial alignment, respectively. Representative snapshots overlaid on the distribution illustrate the two typical local packing modes sampled from these terminal regimes, with the NAC segment highlighted in green and the N- and C-terminal regions shown in blue and red, respectively. These examples show that the ordered NAC segments can form both co-oriented and oppositely oriented pairwise contacts within the condensate. The distribution shows that Hel$_{50}$ enriches collinear NAC packing in both directions rather than producing a single dominant antiparallel population. (B) NAC anti-diagonal contact enrichment, P_{anti}, as a function of cluster size. P_{anti} was calculated as $100 \times (\langle s_{\text{anti}} \rangle / \langle s_{\text{parallel}} \rangle - 1)$, where $\langle s_{\text{anti}} \rangle$ and $\langle s_{\text{parallel}} \rangle$ are the mean smooth contact scores in broad anti-diagonal and parallel-register NAC bands, respectively. Positive values indicate stronger anti-diagonal than parallel-register contacts. The positive mean values in intermediate-sized Hel$_{50}$ clusters are consistent with broad head-to-tail-like NAC registry, although the signal is variable across replicates. Solid lines and shaded bands in (A) indicate the mean and SD, respectively, across three independent replicate trajectories; symbols and error bars in (B) indicate the corresponding mean and SD.

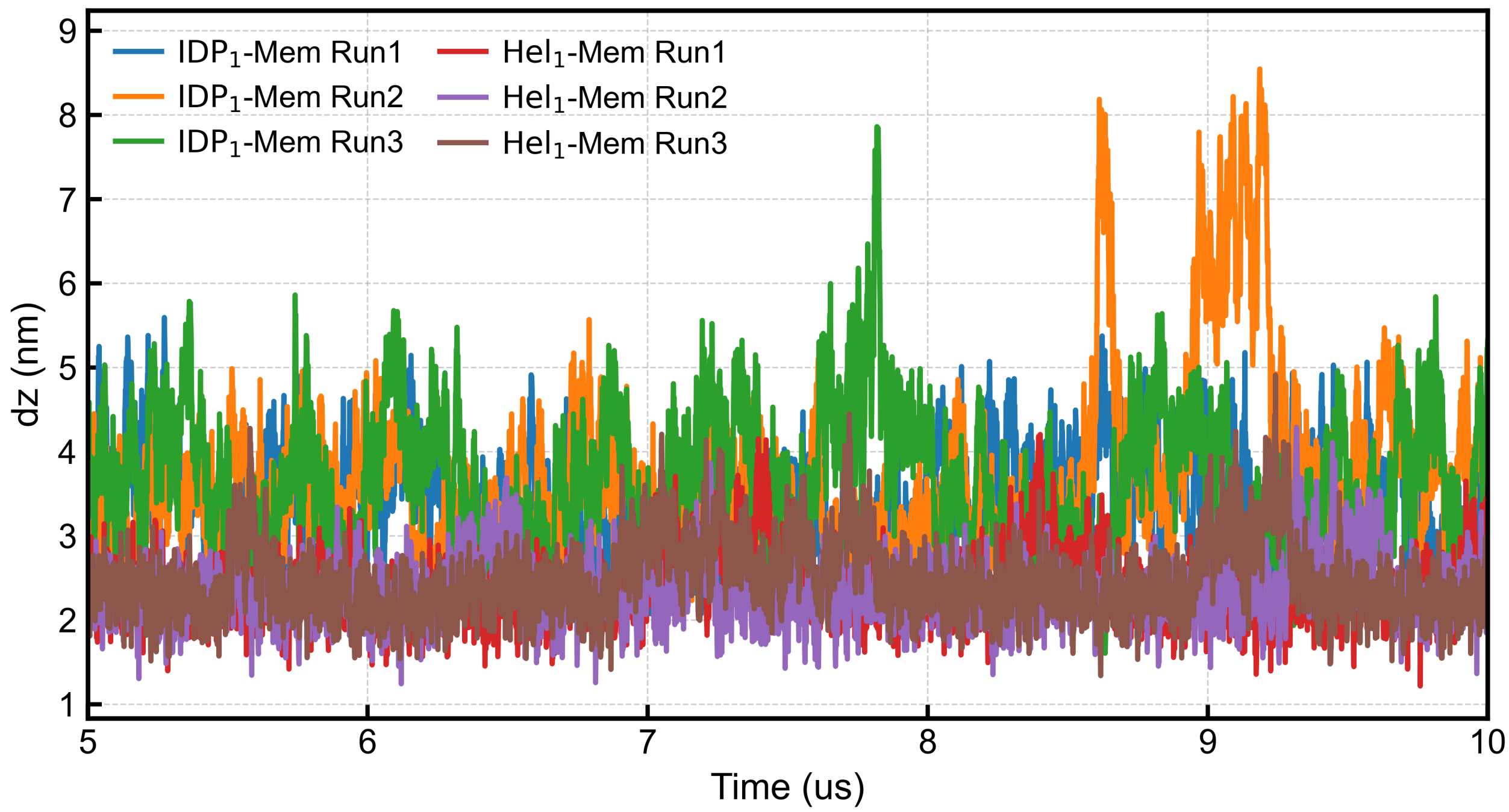

Figure S6: Membrane-normal protein–membrane center-of-mass distance in the single-chain membrane simulations. The distance $d_z = |z_{\text{protein}} - z_{\text{membrane midplane}}|$ is shown during the final 5 μs for three independent replicate trajectories of IDP$_1$-Mem and Hel$_1$-Mem. The lower d_z values for Hel$_1$-Mem indicate closer membrane association.

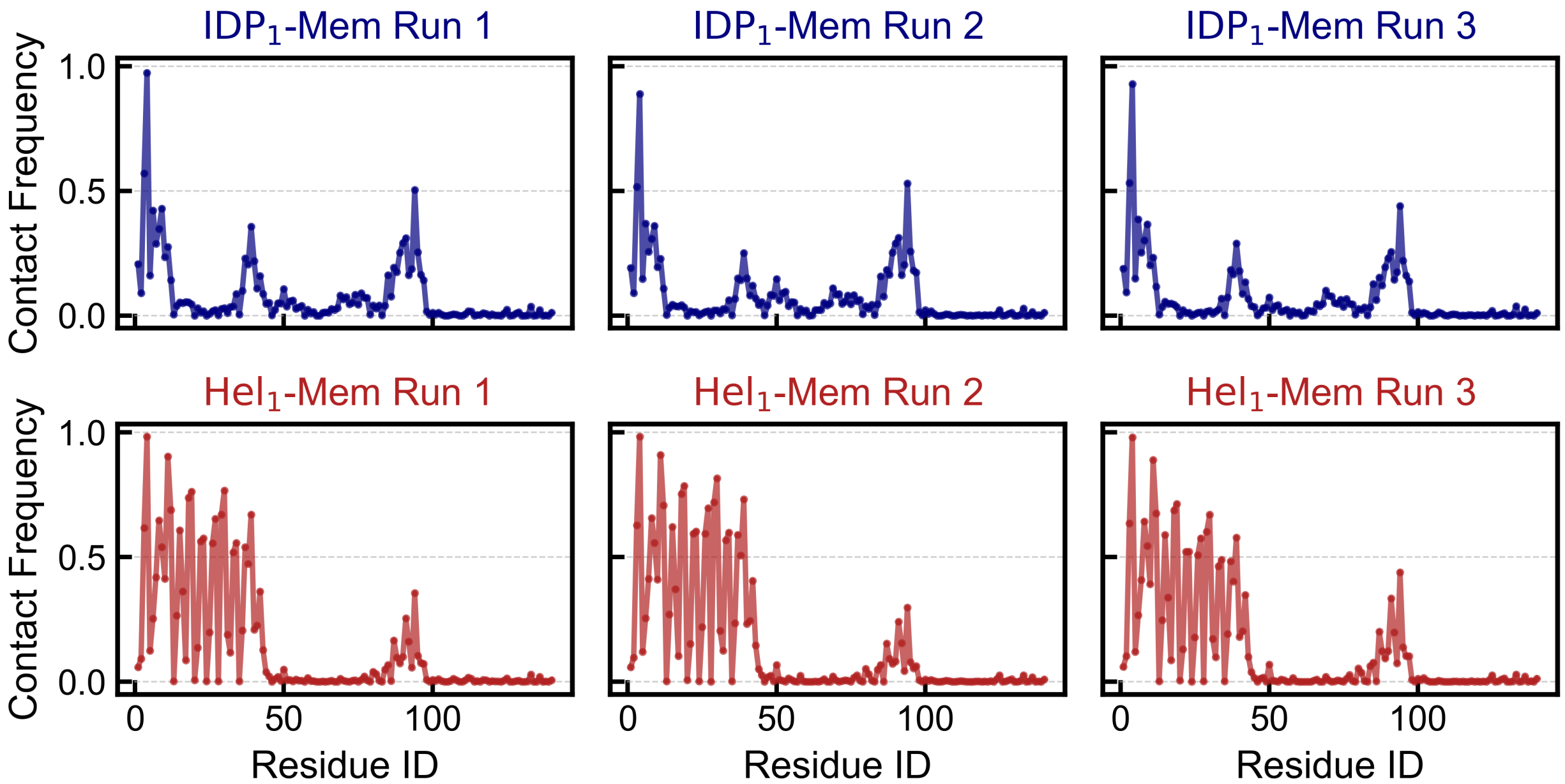

Figure S7: Residue-wise membrane-contact frequency, showing enhanced N-terminal membrane contacts in Hel$_1$-Mem. Three replicates are shown.

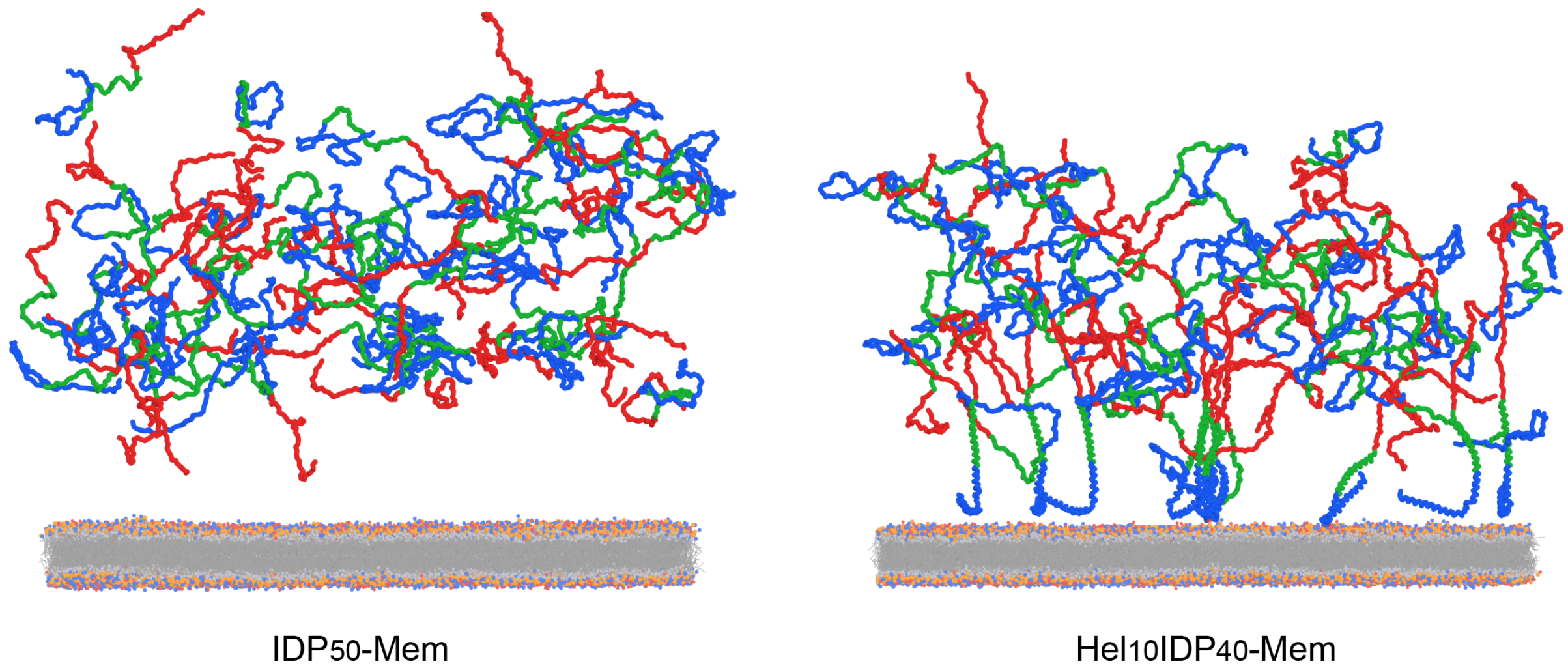

Figure S8: Initial configuration of the IDP_{50}-Mem and $Hel_{10}IDP_{40}$-Mem systems. The first system contains 50 fully disordered chains. The second system contains 40 fully disordered chains and 10 partially helical chains above a POPC:POPG = 7:3 lipid bilayer. The partially helical chains have their N-terminal regions restrained along the z direction to maintain membrane association, while allowing lateral movement in the xy plane.

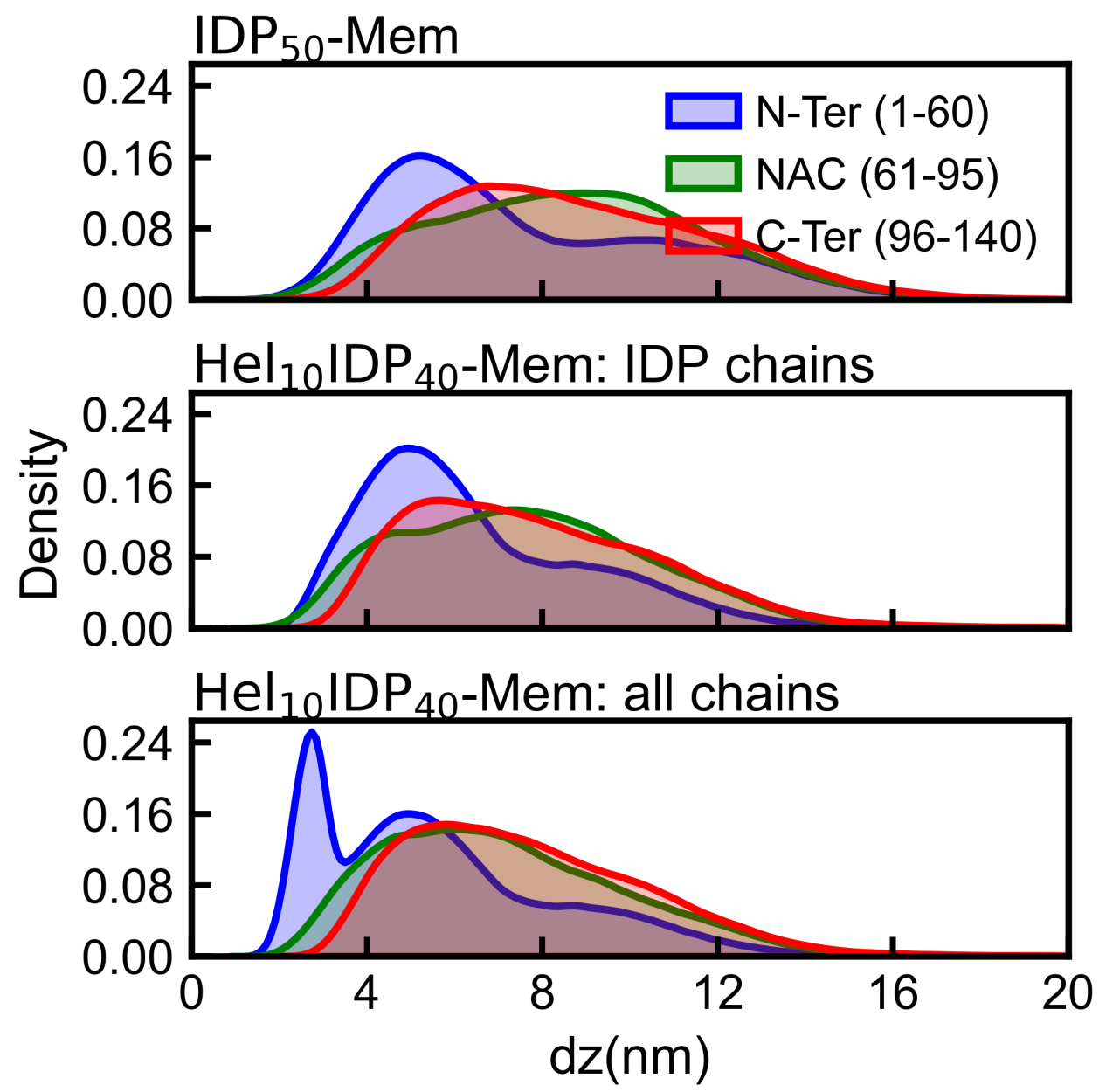

Figure S9: Segment-resolved membrane-normal distributions in membrane-associated multichain systems. Kernel-density distributions show the membrane-normal center-of-mass displacement relative to the membrane midplane, d_z, for the N-terminal region (residues 1–60), NAC region (residues 61–95), and C-terminal region (residues 96–140). IDP_{50}-Mem and the fully disordered chains in $Hel_{10}IDP_{40}$-Mem show preferential N-terminal localization closest to the membrane, whereas the NAC and C-terminal regions extend farther from the bilayer. Including all chains in $Hel_{10}IDP_{40}$-Mem reveals an additional near-membrane N-terminal population from the predefined helical chains, consistent with stronger membrane anchoring by the ordered N-terminal segment.

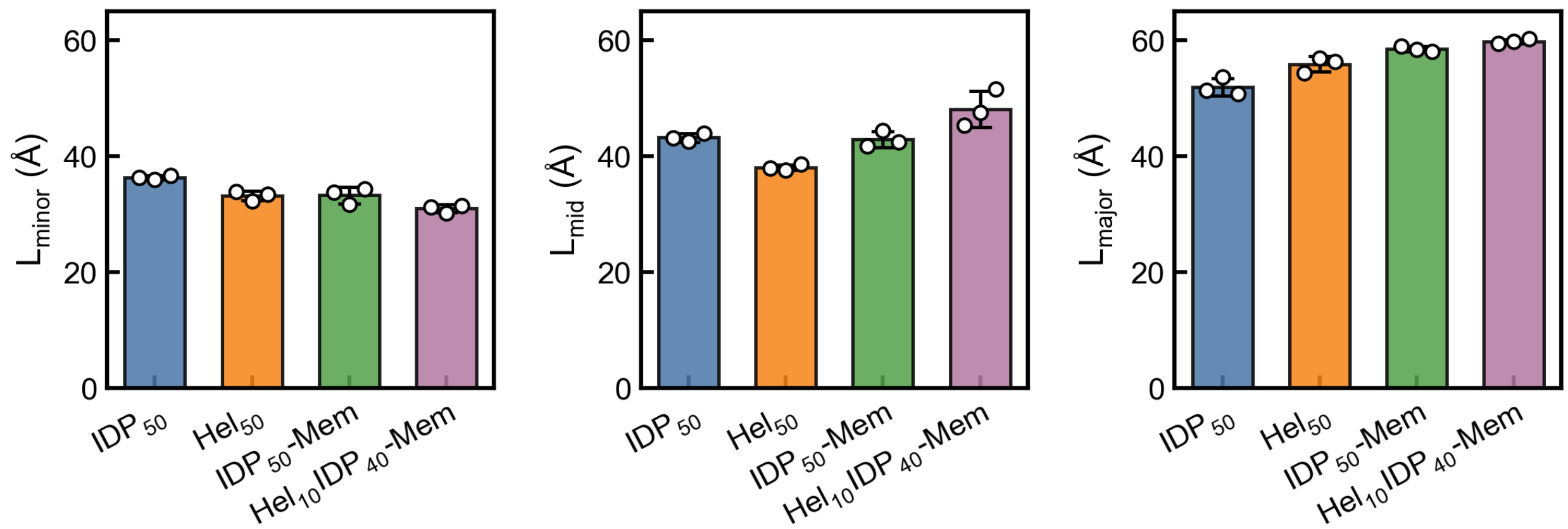

Figure S10: Principal-axis analysis of soluble and membrane-associated condensates. Bars show the three gyration-tensor-derived principal-axis lengths for the largest condensate in IDP_{50}, Hel_{50}, IDP_{50}-Mem, and $Hel_{10}IDP_{40}$-Mem. Open circles denote the three independent replicate trajectories, bars indicate means, and error bars indicate SD across the three trajectories. The separation among the minor, intermediate, and major axes illustrates the increased anisotropy of Hel_{50} and membrane-associated condensates relative to the more isotropic soluble IDP_{50} condensate.

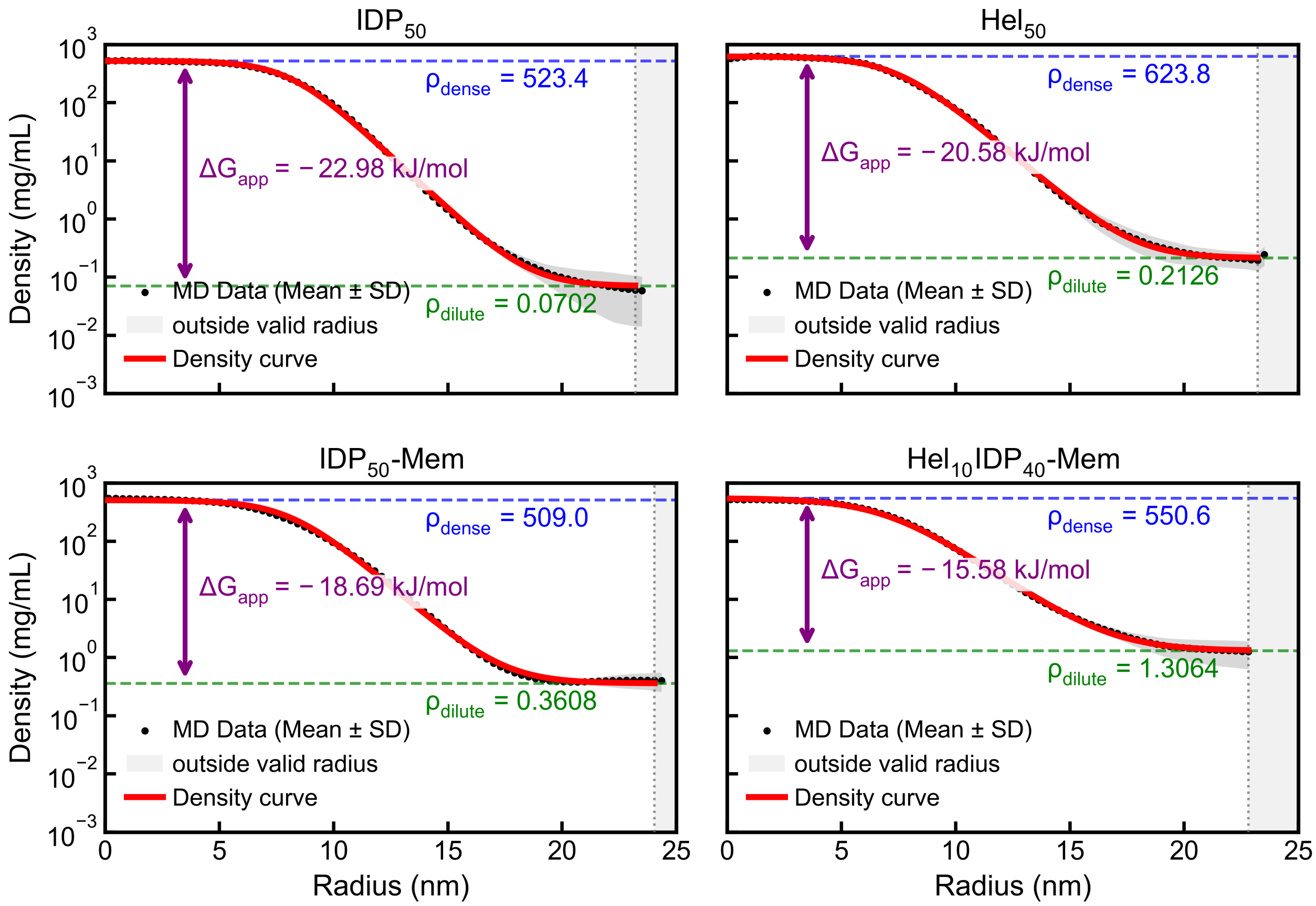

Figure S11: Radial mass-density profiles and hyperbolic-tangent fits used to obtain the fitted density components underlying the apparent transfer free energy, ΔG_{app}. Profiles are shown for IDP_{50}, Hel_{50}, IDP_{50}-Mem, and $\text{Hel}_{10}\text{IDP}_{40}$-Mem after centering each frame on the largest condensate. Black points and gray bands show the MD density profiles as mean $\pm$ SD across three independent replicate trajectories, and red curves show hyperbolic-tangent fits to the replicate-averaged profiles. Blue dashed lines mark the fitted dense-phase plateau density, ρ_{dense}, and green dashed lines mark the fitted dilute-phase plateau density, ρ_{dilute}. Gray shaded regions indicate radial distances outside the maximum valid radial range imposed by the periodic-box geometry. In each analyzed frame, the maximum valid radius was defined as one half of the shortest instantaneous box dimension. The ΔG_{app} values were calculated as $-RT\ln(\rho_{\text{dense}}/\rho_{\text{dilute}})$ from the fitted replicate-averaged profiles and are shown as visual summaries of the interfacial fits. The quantitative values in Fig. 3D were obtained by fitting each replicate trajectory independently and reporting the mean and SD of the three replicate-wise ΔG_{app} estimates.

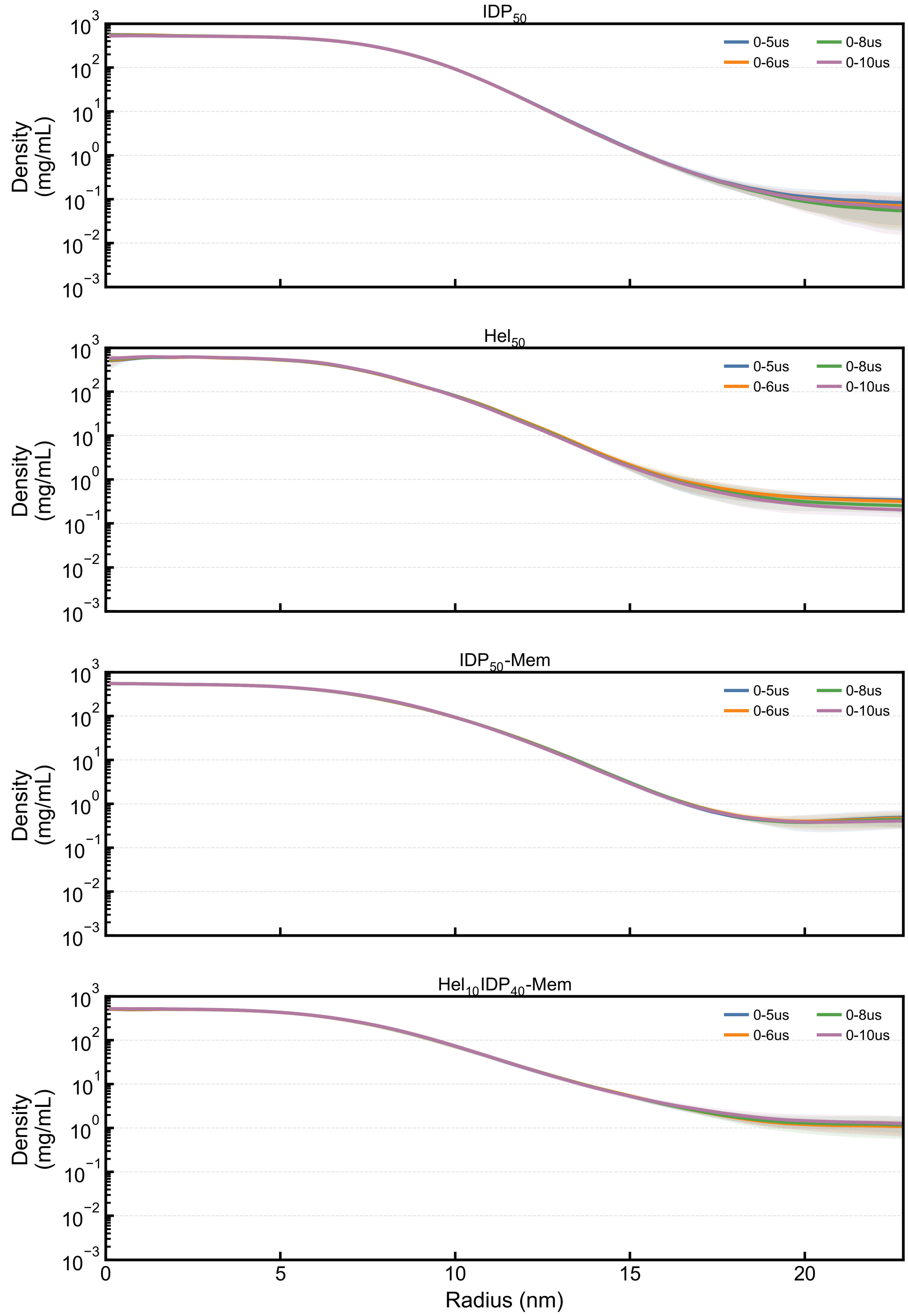

Figure S12: Stability of radial density profiles with increasing sampling length. Radial mass-density profiles of IDP_{50}, Hel_{50}, IDP_{50}-Mem, and $Hel_{10}IDP_{40}$-Mem were calculated over progressively extended cumulative portions of the system-specific late-stage analysis windows. For each system, the beginning of the analysis window was defined as 0 μs, and profiles were evaluated over cumulative intervals of 0–5, 0–6, 0–8, and 0–10 μs. Solid lines indicate the replicate-averaged profiles, and shaded regions indicate the variation across three independent trajectories.

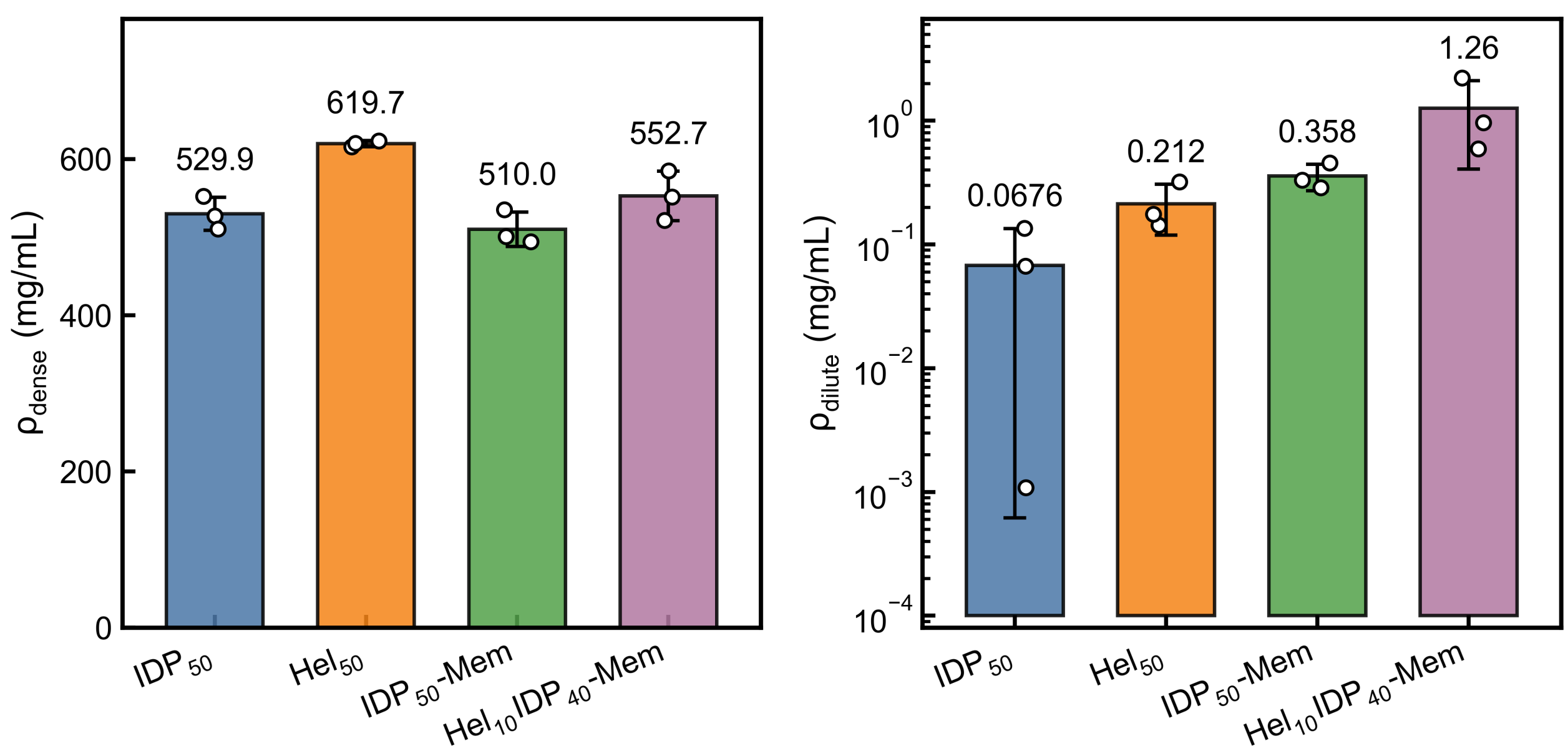

Figure S13: Fitted dense- and dilute-phase plateau densities underlying ΔG_{app}. Bar plots compare ρ_{dense} and ρ_{dilute} obtained from independent hyperbolic-tangent fits to the three replicate trajectories of IDP_{50}, Hel_{50}, IDP_{50}-Mem, and $\mathrm{Hel}_{10}\mathrm{IDP}_{40}$-Mem. Open circles denote the fitted values from the individual replicate trajectories, bars indicate means, and error bars indicate SD across the three replicates. The fitted dense-phase densities remain broadly comparable among systems, whereas ρ_{dilute} increases from soluble IDP_{50} to Hel_{50}, IDP_{50}-Mem, and most strongly $\mathrm{Hel}_{10}\mathrm{IDP}_{40}$-Mem, explaining the progressively weakened apparent partitioning into the largest condensate.

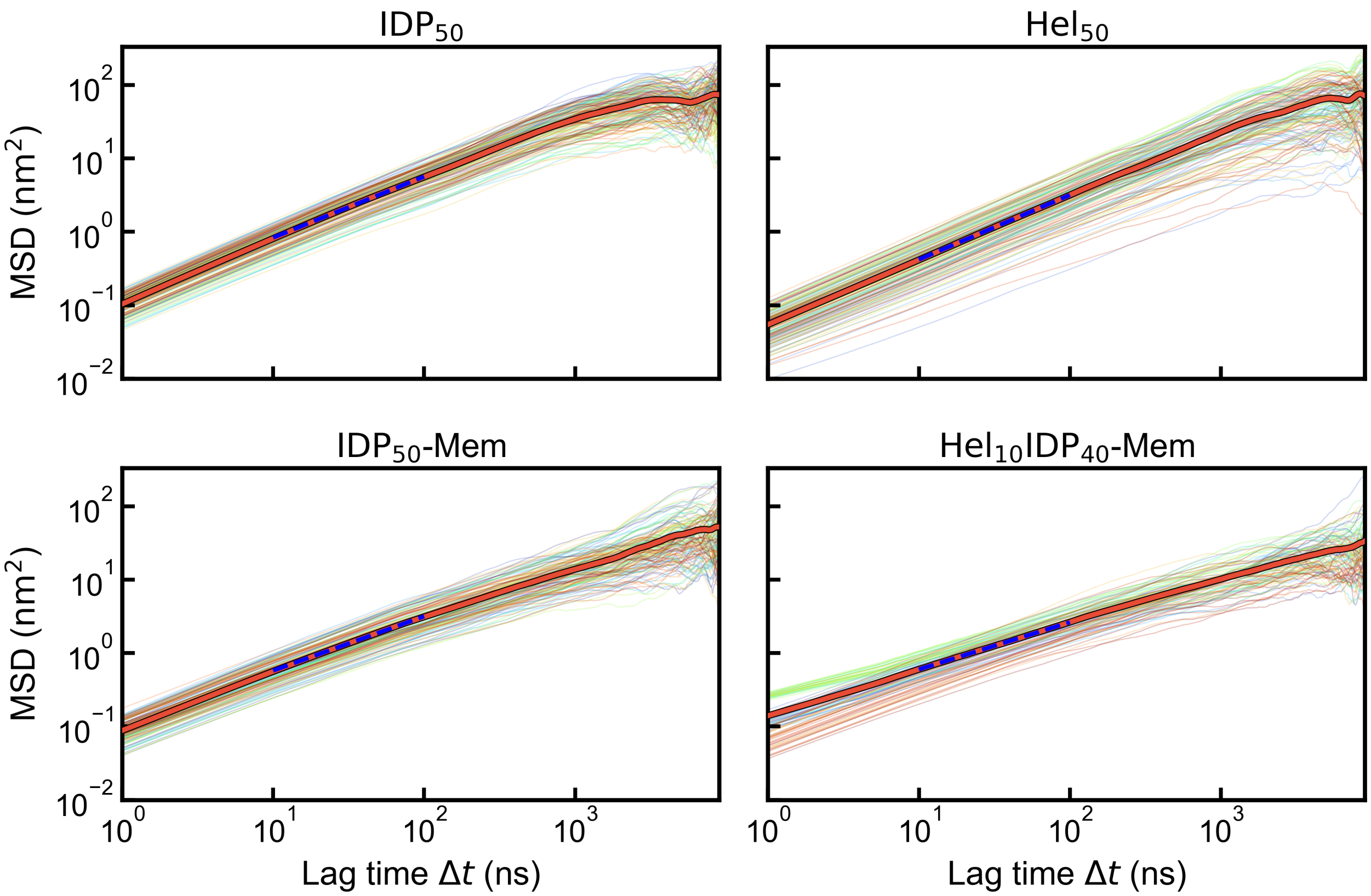

Figure S14: Raw log–log MSD curves used to estimate anomalous diffusion exponents for the IDP$_{50}$, Hel$_{50}$, IDP$_{50}$-Mem, and Hel$_{10}$IDP$_{40}$-Mem condensates. Thin colored curves show individual chain-level MSD traces from chains retained in the largest condensate, and the thick curve shows the ensemble-averaged MSD. The blue dashed lines indicate the power-law fitting region used to obtain α from $\mathrm{MSD}(\tau) \sim \tau^{\alpha}$. The shallower slopes in the membrane-associated systems support the enhanced subdiffusive behavior reported in Fig. 4B.

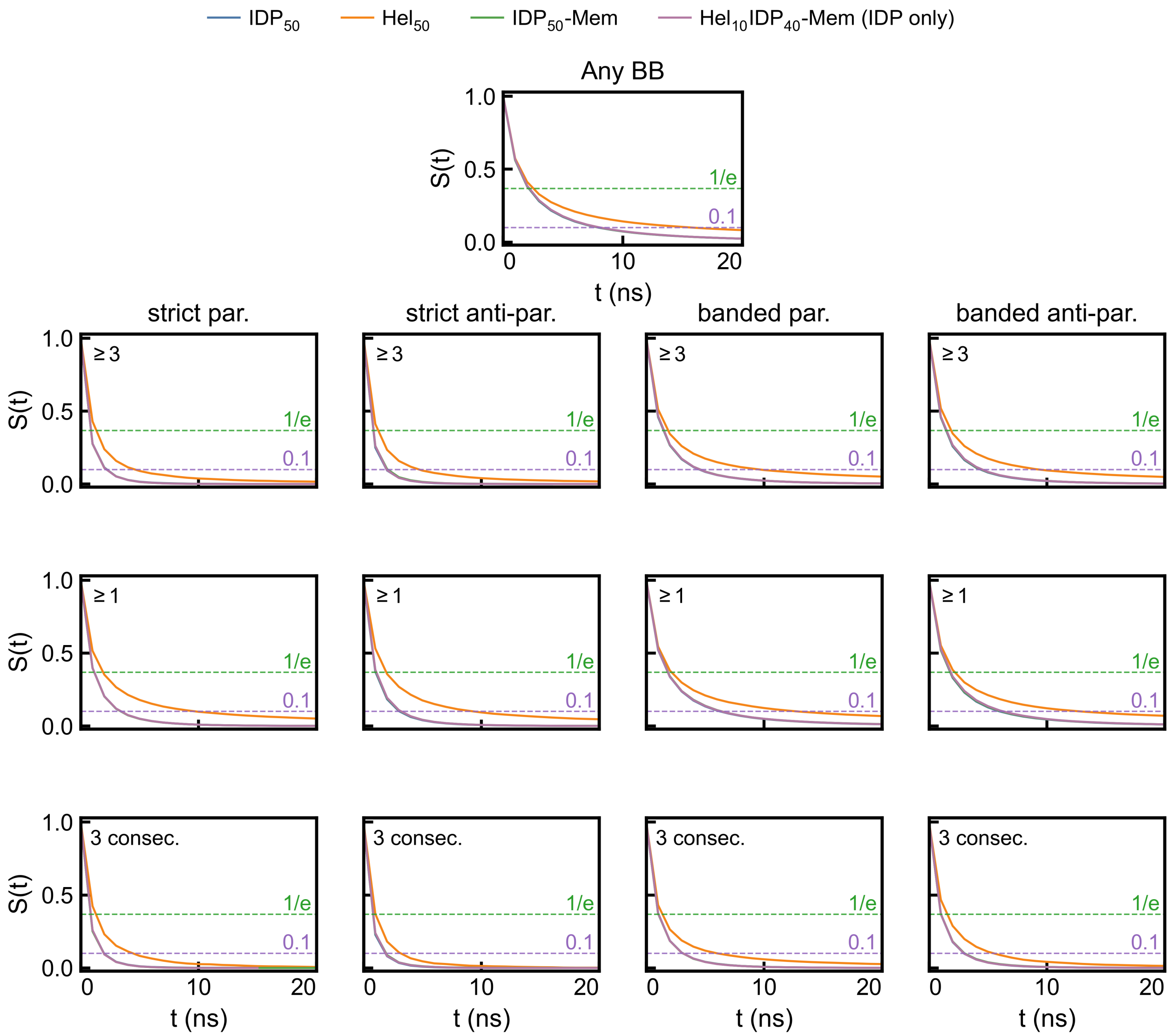

Figure S15: Empirical survival functions for continuous inter-chain NAC contact episodes under the complete hierarchy of contact definitions. $S(t)$ is the fraction of contact episodes that remain continuously present for at least time t, averaged over three independent trajectories. Columns correspond to strict parallel-like contacts (strict par.), strict antiparallel-like contacts (strict anti-par.), banded parallel-like contacts (banded par.), and banded antiparallel-like contacts (banded anti-par.). Rows show any NAC backbone–backbone contact, at least three qualifying NAC residue contacts, at least one qualifying NAC residue contact, and three consecutive qualifying contacts, respectively. The upper panel shows the Any BB–BB definition as a common reference. Green and purple dashed horizontal lines mark $S = 1/e$ and $S = 0.1$, respectively. Soluble Hel_{50} generally exhibits slower contact survival than IDP_{50}, whereas the two membrane-associated systems remain close to soluble IDP_{50} across most contact definitions.

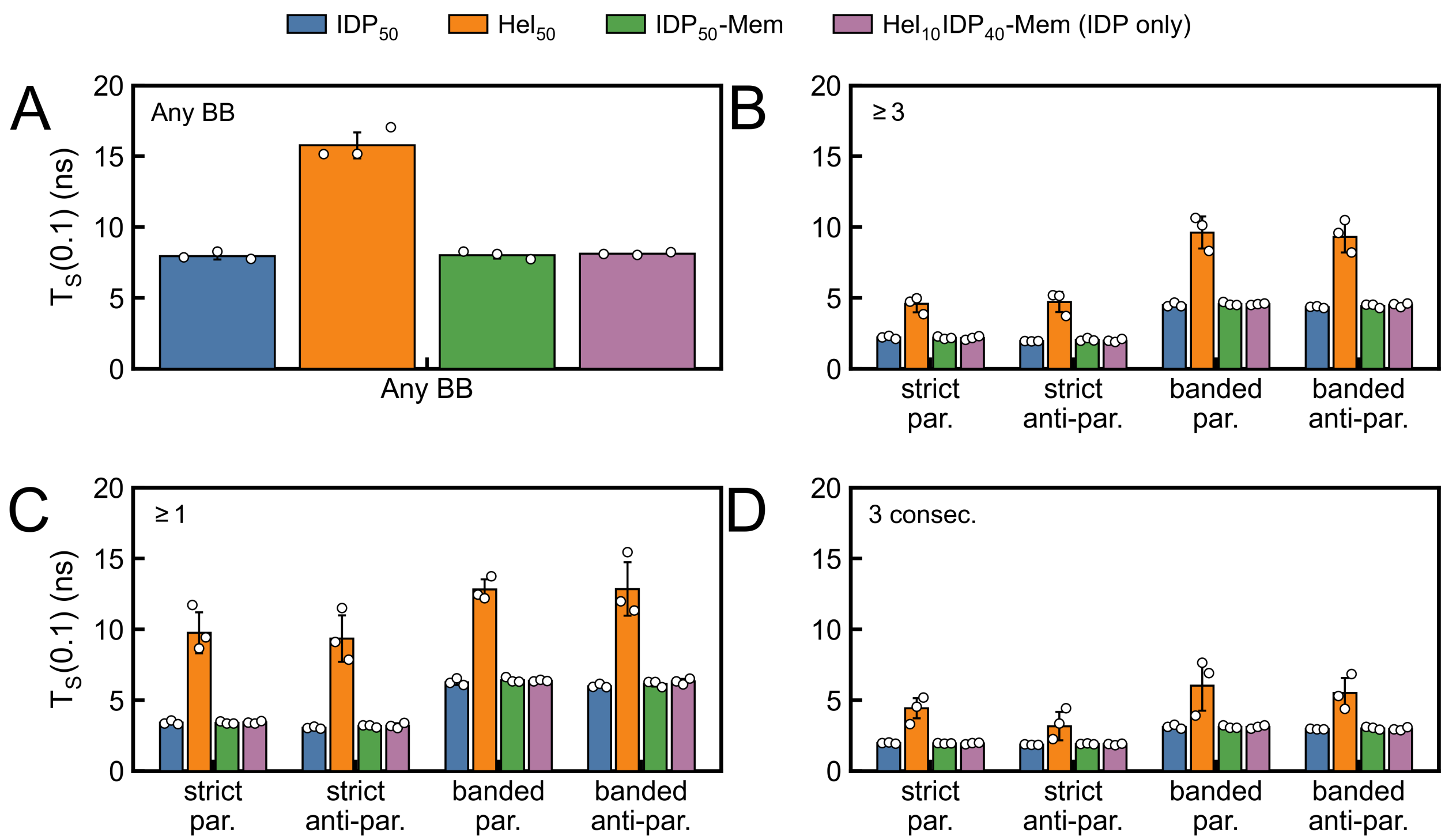

Figure S16: Long-tail continuous NAC contact-survival times extracted at $S(t) = 0.1$. Bars show $T_S(0.1)$, the first time at which the empirical survival probability decreases to 0.1. (A) Any NAC backbone–backbone contact. (B) At least three strict parallel-like, strict antiparallel-like, banded parallel-like, or banded antiparallel-like contacts. (C) At least one contact under the same four registry classes. (D) Three-consecutive-residue strict parallel-like, strict antiparallel-like, banded parallel-like, or banded antiparallel-like contacts. Bars indicate means and error bars indicate SD across three independent trajectories. Soluble Hel_{50} shows the largest $T_S(0.1)$ values, whereas the membrane-associated systems generally remain close to soluble IDP_{50}.

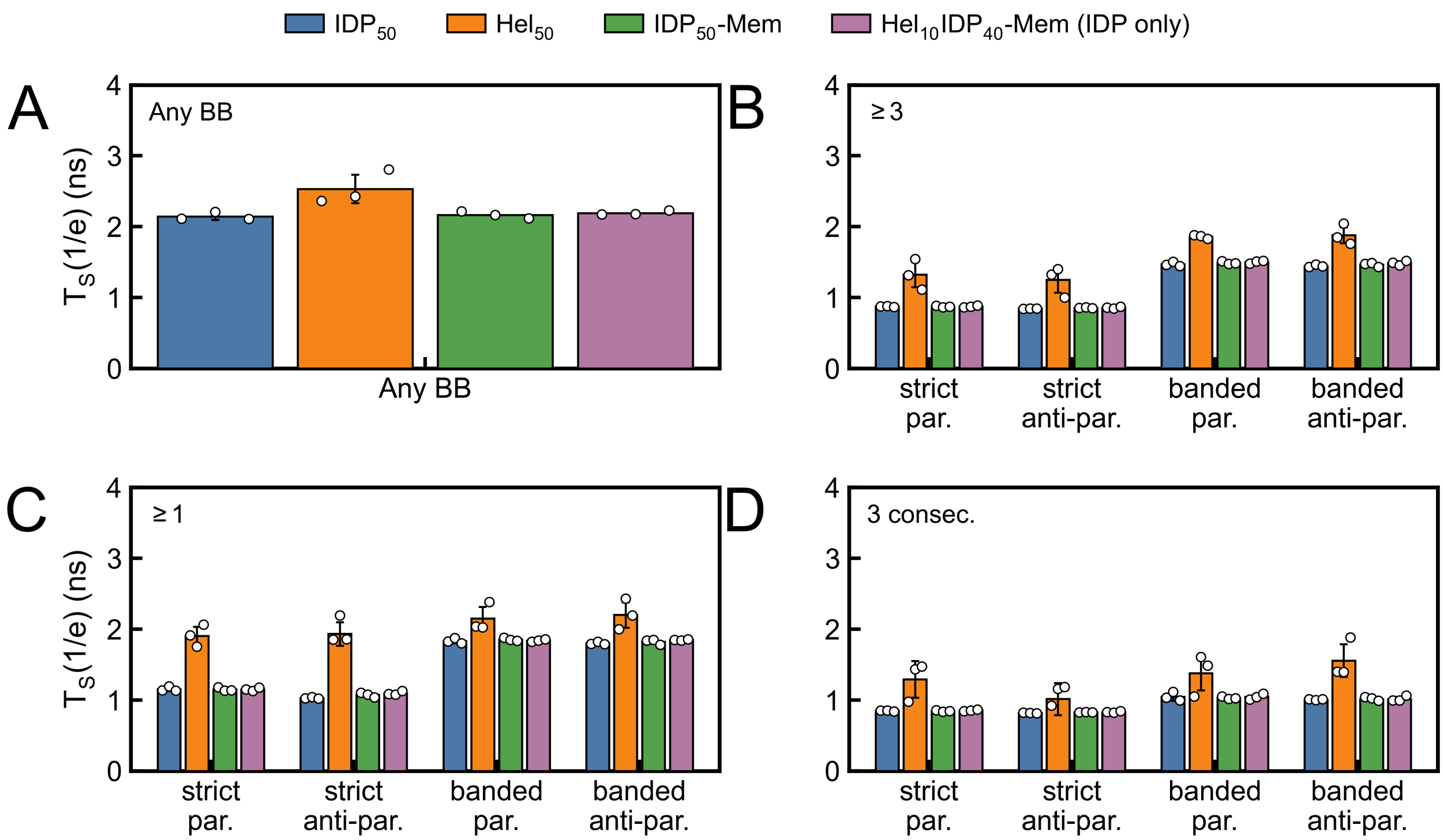

Figure S17: Characteristic continuous NAC contact-survival times extracted at $S(t) = 1/e$. Bars show $T_S(1/e)$, the first time at which the empirical survival probability decreases to $1/e$. (A) Any NAC backbone–backbone contact. (B) At least three strict parallel-like, strict antiparallel-like, banded parallel-like, or banded antiparallel-like contacts. (C) At least one contact under the same four registry classes. (D) Three-consecutive-residue strict parallel-like, strict antiparallel-like, banded parallel-like, or banded antiparallel-like contacts. Bars indicate means and error bars indicate SD across three independent trajectories. Predefined ordering in soluble Hel_{50} increases the characteristic contact lifetime, whereas neither membrane-associated system exhibits a systematic increase relative to soluble IDP_{50}.

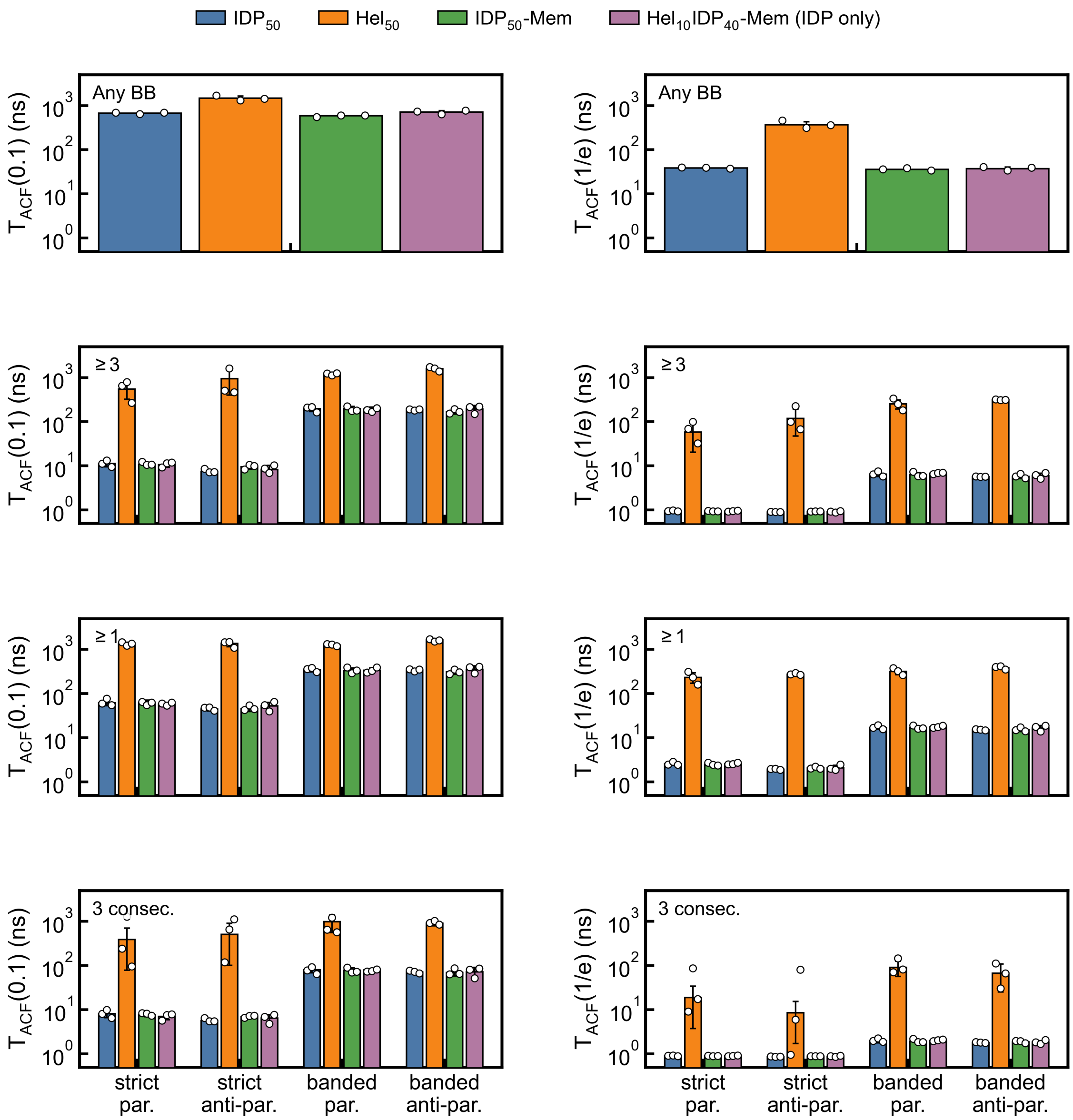

Figure S18: Characteristic NAC contact-state autocorrelation times under the complete hierarchy of contact definitions. The left and right columns show $T_{\mathrm{ACF}}(0.1)$ and $T_{\mathrm{ACF}}(1/e)$, respectively, defined as the first times at which the normalized contact autocorrelation function decreases to 0.1 and $1/e$. From top to bottom, the rows show any NAC backbone–backbone contact, at least three qualifying NAC residue contacts, at least one qualifying NAC residue contact, and three consecutive qualifying contacts. Within the lower three rows, results are shown for the strict parallel-like, strict antiparallel-like, banded parallel-like, and banded antiparallel-like registry classes. The ordinate is logarithmic. Open circles show values from individual trajectories; bars and error bars show the mean and SD across the three independent trajectories. Soluble Hel_{50} exhibits substantially slower contact-state decorrelation than the other systems, whereas the membrane-associated systems generally remain close to soluble IDP_{50}.

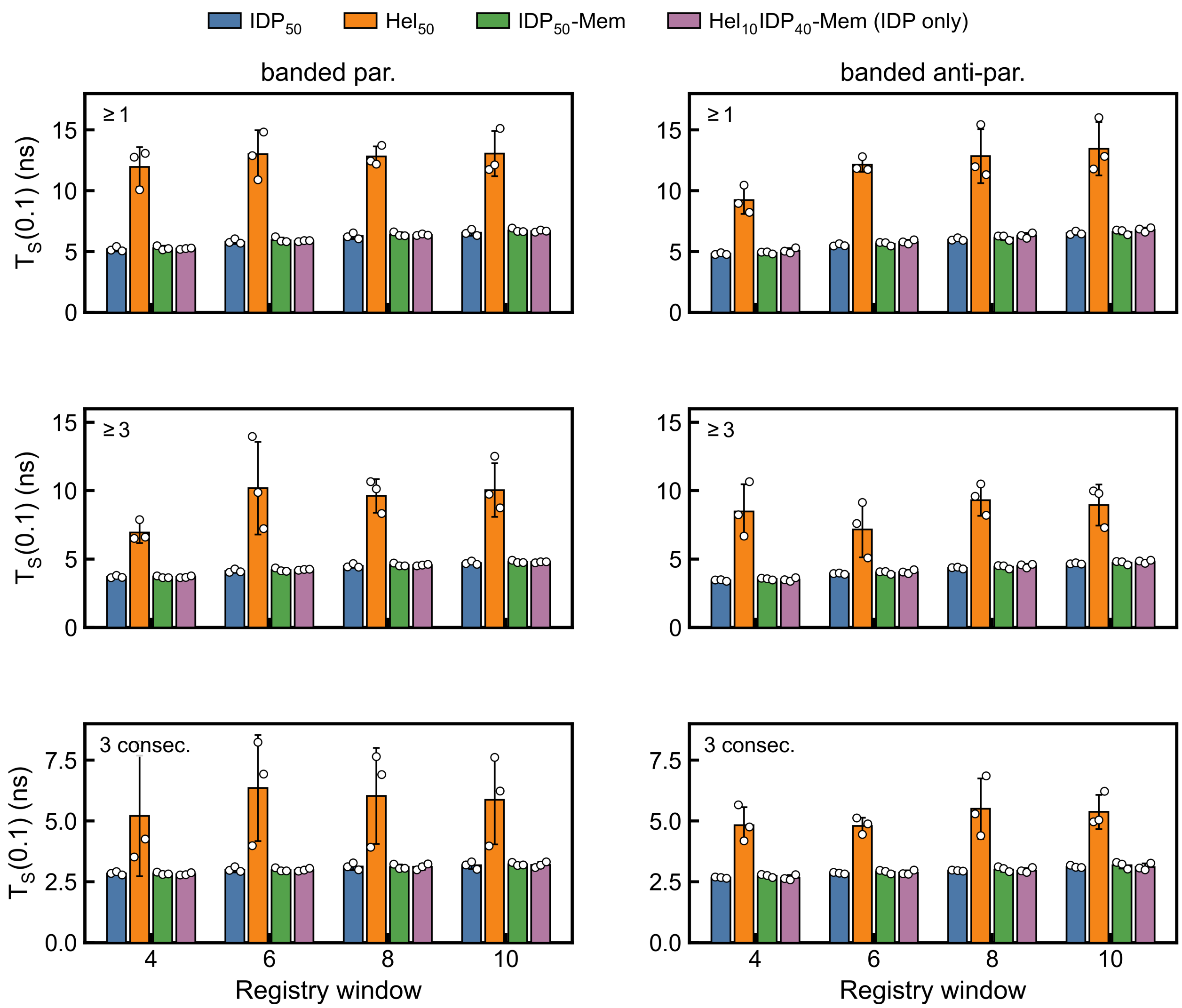

Figure S19: Sensitivity of NAC contact-survival times to the allowed registry window. Characteristic contact-survival times, $T_S(0.1)$, were recalculated for banded parallel-like (left) and banded antiparallel-like (right) NAC contacts using registry windows of 4, 6, 8, and 10 residues. From top to bottom, the contact criteria require at least one qualifying contact, at least three qualifying contacts, or at least three consecutive qualifying contacts. For $Hel_{10}IDP_{40}$-Mem, the analysis corresponds to contacts between the fully disordered chains (IDP–IDP pairs). Increasing the registry window generally increased the absolute contact-survival times by making the registry definition more permissive, while the major system-dependent trend was preserved across the tested window sizes, with Hel_{50} showing consistently longer-lived NAC contacts than the other systems. Bars indicate the mean $T_S(0.1)$ across three independent trajectories, error bars indicate SD, and open circles show individual replicate values.

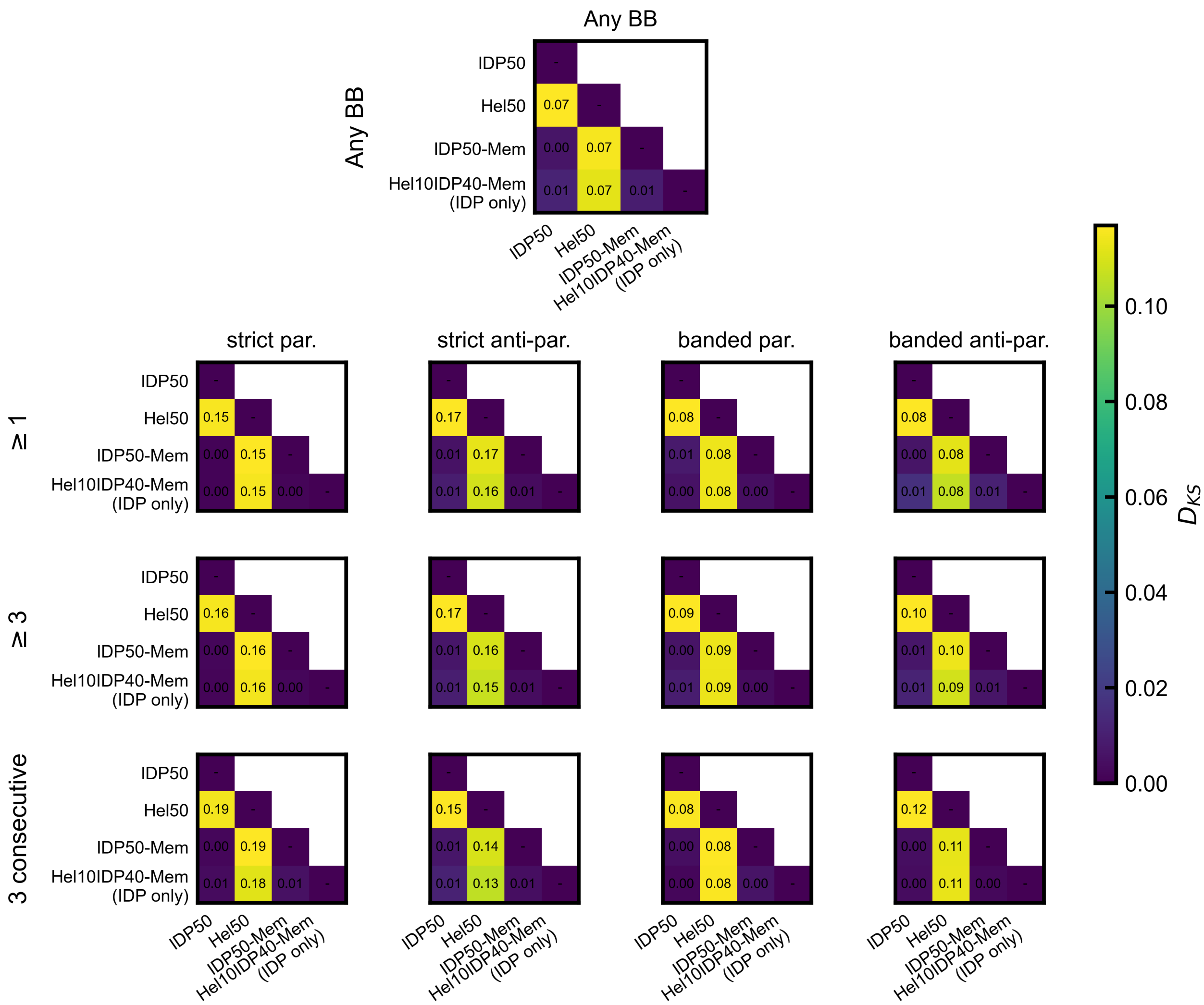

Figure S20: Pairwise Kolmogorov–Smirnov comparison of complete NAC contact-lifetime distributions. Pairwise Kolmogorov–Smirnov statistics, D_{KS}, were calculated from the empirical NAC contact-lifetime distributions of IDP_{50}, Hel_{50}, IDP_{50}-Mem, and $\mathrm{Hel}_{10}\mathrm{IDP}_{40}$-Mem (IDP–IDP pairs). The upper panel shows the Any BB contact definition. The remaining panels show strict parallel-like, strict antiparallel-like, banded parallel-like, and banded antiparallel-like contacts for criteria requiring at least one contact (top row), at least three contacts (middle row), or at least three consecutive contacts (bottom row). Each matrix reports the lower-triangular pairwise D_{KS} values; larger values indicate greater separation between the two complete lifetime distributions. Comparisons involving Hel_{50} consistently showed larger D_{KS} values, whereas the distributions of IDP_{50} and the membrane-associated disordered-chain populations remained much more similar.

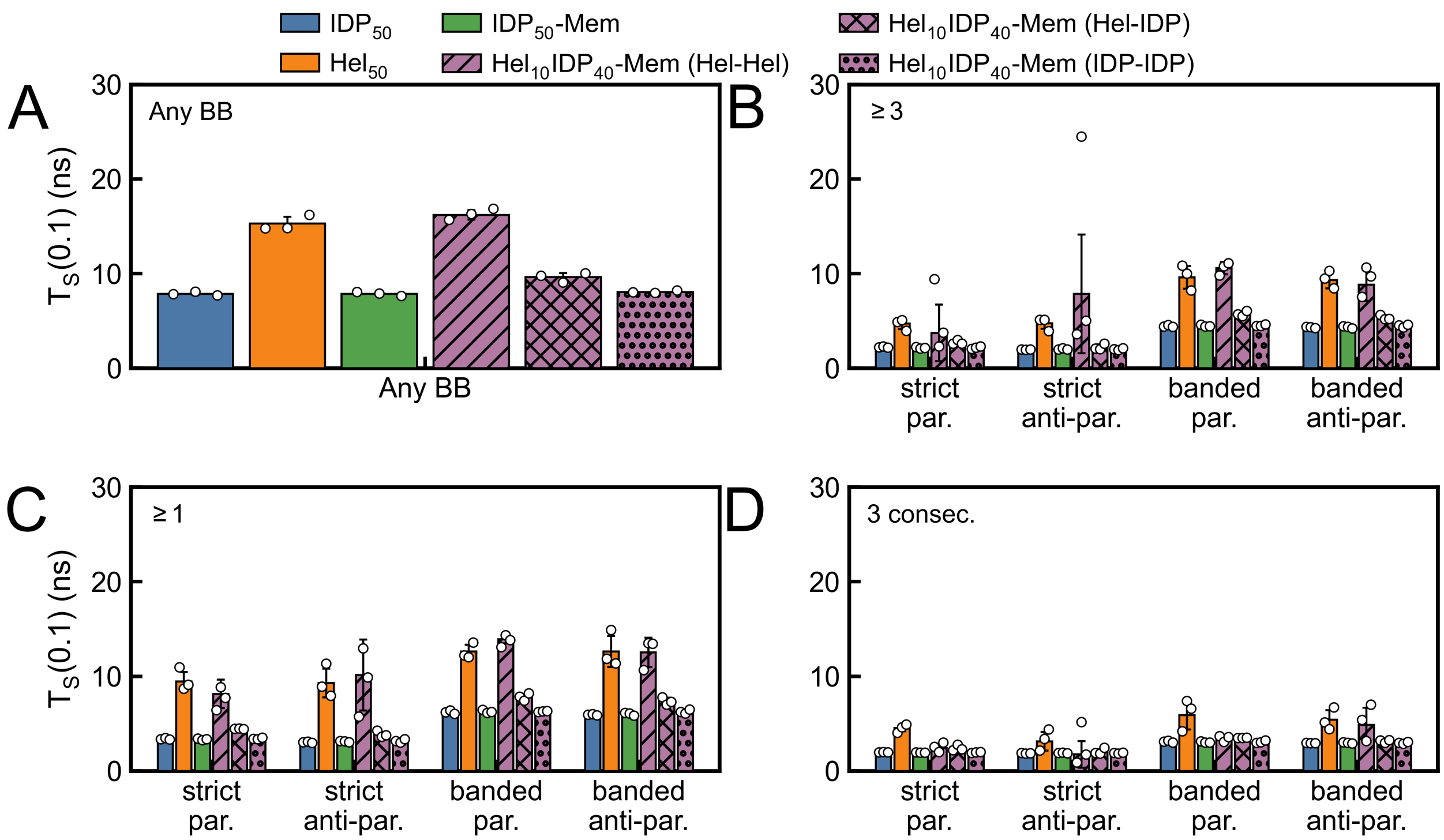

Figure S21: Pair-type-resolved NAC contact persistence in the mixed $Hel_{10}IDP_{40}$-Mem system. Characteristic continuous NAC contact-survival times, $T_S(0.1)$, were compared among IDP_{50}, Hel_{50}, IDP_{50}-Mem, and the Hel–Hel, Hel–IDP, and IDP–IDP pair classes within $Hel_{10}IDP_{40}$-Mem. (A) Any NAC backbone–backbone contact. (B) Contact definitions requiring at least three qualifying contacts for strict parallel-like, strict antiparallel-like, banded parallel-like, and banded antiparallel-like registries. (C) Corresponding definitions requiring at least one qualifying contact. (D) More restrictive definitions requiring at least three consecutive residues to maintain strict or banded parallel-like or antiparallel-like registry. Bars indicate the mean $T_S(0.1)$ across three independent trajectories, error bars indicate SD, and open circles show individual replicate values.
